# Retention of duplicated long-wavelength opsins in mosquito lineages by positive selection and differential expression

**DOI:** 10.1186/s12862-017-0910-6

**Published:** 2017-03-21

**Authors:** Gloria I. Giraldo-Calderón, Michael J. Zanis, Catherine A. Hill

**Affiliations:** 1 Department of Entomology, Purdue University, West Lafayette, IN 47907-2089 USA; 2 Department of Botany and Plant Pathology, Purdue University, West Lafayette, IN 47907-2089 USA; 3 Purdue Institute of Inflammation, Immunology and Infectious Disease, Purdue University, West Lafayette, IN 47907-2089 USA; 4 Present Address: Department of Biological Sciences, University of Notre Dame, Notre Dame, IN 46556 USA; 5 Present Address: Department of Biology, Seattle University, Seattle, WA 98122 USA

**Keywords:** Mosquito, Opsin, Long-wavelength, Gene duplication, Positive selection, Differential expression, Conserved non-coding sequences, Vision

## Abstract

**Background:**

Opsins are light sensitive receptors associated with visual processes. Insects typically possess opsins that are stimulated by ultraviolet, short and long wavelength (LW) radiation. Six putative LW-sensitive opsins predicted in the yellow fever mosquito, *Aedes aegypti* and malaria mosquito, *Anopheles gambiae,* and eight in the southern house mosquito, *Culex quinquefasciatus,* suggest gene expansion in the Family Culicidae (mosquitoes) relative to other insects. Here we report the first detailed molecular and evolutionary analyses of LW opsins in three mosquito vectors, with a goal to understanding the molecular basis of opsin-mediated visual processes that could be exploited for mosquito control.

**Results:**

Time of divergence estimates suggest that the mosquito LW opsins originated from 18 or 19 duplication events between 166.9/197.5 to 1.07/0.94 million years ago (MY) and that these likely occurred following the predicted divergence of the lineages Anophelinae and Culicinae 145–226 MY. Fitmodel analyses identified nine amino acid residues in the LW opsins that may be under positive selection. Of these, eight amino acids occur in the N and C termini and are shared among all three species, and one residue in TMIII was unique to culicine species. Alignment of 5′ non-coding regions revealed potential Conserved Non-coding Sequences (CNS) and transcription factor binding sites (TFBS) in seven pairs of LW opsin paralogs.

**Conclusions:**

Our analyses suggest opsin gene duplication and residues possibly associated with spectral tuning of LW-sensitive photoreceptors. We explore two mechanisms - positive selection and differential expression mediated by regulatory units in CNS – that may have contributed to the retention of LW opsin genes in Culicinae and Anophelinae. We discuss the evolution of mosquito LW opsins in the context of major Earth events and possible adaptation of mosquitoes to LW-dominated photo environments, and implications for mosquito control strategies based on disrupting vision-mediated behaviors.

**Electronic supplementary material:**

The online version of this article (doi:10.1186/s12862-017-0910-6) contains supplementary material, which is available to authorized users.

## Background

The genomes of eukaryotes typically comprise a high percentage of duplicated genes. It has been proposed that gene duplication is the major mechanism for the origin of new gene function and that the refashioning of duplicate genes is a major contributor to the origin of adaptive evolutionary novelties [1, 2]. Gene duplication may originate from unequal crossing over, retro-transposition, segmental duplication and chromosomal or genome duplication [3]. Force et al. [4] proposed several theories to explain the retention of duplicate gene copies in the genome wherein the fate of each copy may depend on mutations that occur in both coding and regulatory regions. The accumulation of mutations in one copy may eventually render the gene non-functional (*i.e*., a pseudogene). Degenerative mutations may accumulate in the regulatory regions of gene copies that are separately capable of performing a distinct ancestral function (subfunctionalization) or one copy may retain the original function with the second copy acquiring a new function by the retention of beneficial mutations (neofunctionalization).

Opsins initiate the photon-induced signaling cascade in vertebrates and invertebrates, and are members of the G protein-coupled receptor (GPCR) family, characterized by seven trans-membrane domains (TMDs I - VII). Insects typically possess three classes of visual opsins that are sensitive to ultraviolet (UV, λ_max_ 300–400 nm), short (SW, λ_max_ 400–500 nm) and long (LW, λ_max_ 500–600 nm) wavelengths. Additionally, some insects have red (λ_max_ >565 nm) sensitive receptors [5]. Visual opsins are expressed in the rhabdomere of the ommatidia, the major structural unit of the arthropod compound eye. Non visual opsins have also been identified and include the *Apis mellifera* (honey bee) pteropsin identified in the bee brain, suggesting a possible function in extra-retinal detection of light and the regulation of circadian rhythm [6–9]. The functions of the *Drosophila melanogaster* (fruit fly) opsin Rh7 [10] and the RGR-like and arthropsins identified in *Daphnia pulex* (common water flea) have not been determined.

Mosquitoes (Order Diptera, Family Culicidae) are one of the most important arthropod groups affecting human and animal health [11]. The assembled genomes of the yellow fever mosquito *Aedes aegypti*, the malaria mosquito *Anopheles gambiae* and the southern house mosquito *Culex quinquefasciatus* [12–14] provide an opportunity to investigate the molecular evolution of opsin genes in three mosquito taxa representing the lineages Culicinae (includes *Ae. aegytpi* and *Cx. quinquefasciatus*) and Anophelinae (includes *An. gambiae*). It is predicted that the Culicinae and Anophelinae diverged approximately 145–200 million years ago (MY) [15] or 226 MY [16]. Species comprising these lineages transmit a variety of parasites and pathogens of medical and veterinary significance [17] and exhibit markedly different behavioral periodicities. *Aedes aegypti* is a diurnal mosquito, while *An. gambiae* and *Cx. quinquefasciatus* exhibit nocturnal and crepuscular behaviors [18].

Expansions in the genes coding for LW opsins have been noted in a number of invertebrates, including insects. The fruitfly, *D. melanogaster* possesses three LW opsin genes [19] and the butterflies *Papillio xuthus* and *Papillio glaucus* have three and four genes, respectively [20, 21]. Examples of duplication in LW opsin genes have been observed in the aquatic crustacean, *Daphnia pulex,* which has 25 LW opsin genes [22], the stomatopods, *Gonodactylus smithii, Neogonodactylus oerstedii, Odontodactylus scyllarus, Coronis scolopendra* and *Squilla empusa*, which each possess 6 LW opsin genes [23], and the dragonflies *Anax parthenope, Anotogaster sieboldii, Asiagomphus melaenops, Epiophlebia superstes, Indolestes peregrinus, Ischnura asiatica, Macromia amphigena, Mnais costalis, Orthetrum albistylum, Somatochlora uchidai, Sympetrum frequens* and *Tanypteryx pryeri* which have 8–21 LW opsin genes [24]. It has been proposed that the opsin expansion observed in *Daphnia* and other aquatic lineages may be influenced by more complex light regimes associated with aquatic environments [22]. The study of Futahashi et al. [24] also suggests an association of LW opsins with aquatic habitat.

Previously, we identified 10 and 11 putative opsins in *Ae. aegypti* [13] and *An. gambiae* [25], respectively, and an expansion of putative LW opsin genes in both species which possess six gene copies as compared to other insects that typically have between one to four LW opsins. Five *An. gambiae* LW opsins (*AgGPRop1, 3–6*) are tandemly arrayed within a 90 kb region on chromosome 2R and are separated from a sixth, and presumably more ancestral LW opsin (*AgGPRop7*), by approximately 3 Mb [25]. The density of genes and segmental duplications as well as the high GC content of 2R (54% as compared to 45% on 2 L) is suggestive of a high recombination rate, a phenomenon typically associated with gene duplication [26]. More recently, 13 opsin genes were found in the *Cx. quinquefasciatus* genome, although their wavelength sensitivity was not predicted [14].

Interest in novel strategies to control mosquitoes is high due to the emergence and re-emergence of arboviral diseases such as dengue [27], yellow fever [28], chikungunya and Zika [29] and the failure of traditional drug and insecticide control. Opsin-mediated processes could be targeted to disrupt mosquito mating, host finding and oviposition. An improved understanding of these processes could also benefit the design of new mosquito traps, deterrent devices [30–34] and genetic control strategies. Here, we present detailed molecular evolutionary analyses of the LW opsins in *Ae. aegypti, An. gambiae* and *Cx. quinquefasciatus* as a first step toward understanding the molecular basis of opsin-mediated visual processes that could be exploited for mosquito control. Phylogenetic analyses suggest that culicine and anopheline mosquitoes possess orthologs of invertebrate LW, SW and UV–sensitive opsins, extra-retinal pteropsins, and the *D. melanogaster* Rh7. We predicted six, six and eight putative LW opsins in *Ae. aegypti*, *An. gambiae*, *Cx. quinquefasciatus,* respectively, indicative of an expansion of these genes in the Culicidae relative to other insects. Molecular evolutionary analyses suggest that mosquito LW opsins originated from 18 or 19 duplication events that occurred between 189.87 to <1 million years ago (MY). The genes for ten LW opsins (*AaGPRop2-5, AgGPRop3-4 and CqGPRop5-9*) may have been retained through a combination of positive selection and coordinated regulation (i.e., acquisition of mutations in the regulatory units in Conserved Non-coding Sequences, CNS). Of the remaining mosquito opsin genes, five may have been retained through positive selection (*AaGPRop1, AgGPRop1, op6 and CqGPRop10 and 13*), and one via coordinated regulation (*CqGPRop7*). Four LW opsins lack evidence of either positive selection or coordinated regulation (*AaGPRop7, AgGPRop5 and op7 and CqGPRop1*).

## Results

Genes for 10, 11 and 13 opsins were manually annotated in the genomes of *Ae. aegypti*, *An. gambiae* and *Cx. quinquefasciatus*, respectively and results are summarized in Table 1. Opsin protein sequences are provided in the Additional file 1 and are also available from VectorBase and GenBank. The protein sequences for all opsins (except *Aa*GPRop9_2, *Ag*GPRop9 and *Cq*GPRop10) contain a predicted initiation methionine, a stop codon, three extracellular (EL) and three intracellular loops (IL), and seven TM domains. Excluding the incomplete models above, the mosquito opsins vary in length from 372 to 502 amino acids in *Ae. aegypti* (av. 393), 370*–*463 in *An. gambiae* (av. 397)*,* and 368–466 amino acids in *Cx. quinquefasciatus* (av. 385)*.* We detected a single amino acid substitution between *Aa*GPRop4 (356 T) and op5 (356S). Of note, *AgGPRop2* first reported in Hill et al. [25] may reflect an erroneous prediction based on an earlier version of the *An. gambiae* assembly. *AaGPRop9_2* is a truncated gene model identical to the first exon of *AaGPRop9_1* from nucleotides 1 to 233, except for a non-synonymous C/A substitution at nucleotide 62 (P21H) and a synonymous T/C substitution at nucleotide 213. We were unable to identify the second exon of *AaGPRop9_2*. The third intracellular loop (IL3) which is likely important for G protein interaction, is 40 amino acids in length in all mosquito opsins except for *Cq*GPRop10 and the presumably more ancestral opsins *Aa*GPRop7, *Ag*GPRop7 and *Cq*GPRop1 where the loop is predicted to be 38, 37, 39 and 39 amino acids in length, respectively*.*

**Table 1:** Summary of *Ae. aegypti, An. gambiae* and *Cx. quinquefasciatus* opsin genes

Gene^a^	NCBI accession no.	VectorBase gene ID	Supercontig or Scaffold	Base pair range on Supercontig/Scaffold	No. amino acids	No. exons	Expression evidence (transcript, peptide)^b^	Predicted wavelength sensitivity range
*Ae. aegypti*								
*AaGPRop1*	XM_001651947.2	AAEL006498	1.208	1,715,269–1,716,390	373	1	t, -	Long
*AaGPRop2*	XM_001657569.2	AAEL006259	1.197	948,069–949,187	372	1	t, -	Long
*AaGPRop3*	XM_001651948.2	AAEL006484	1.208	1,726,735–1,727,928	373	2	t, -	Long
*AaGPRop4*	XM_001651116.2	AAEL005621	1.165	1,775,297–1,776,430	377	1	t, -	Long
*AaGPRop5*	XM_001651117.2	AAEL005625	1.165	1,782,454–1,783,587	377	1	t, -	Long
*AaGPRop7*	XM_001652675.2	AAEL007389	1.252	485,115–486,311	379	2	t, -	Long
*AaGPRop8*	XM_001653816.2	AAEL009615	1.412	271,590–320,188	381	5	t, -	Ultraviolet
*AaGPRop9_1*	XM_001662932.2	AAEL003035	1.75	1,344,859–1,358,612	380	2	t, -	Short
*AaGPRop9_2* ^c^	XM_011494686.1	AAEL003035	1.9	1,914,223–1,914,456	77	1	N.A.	N.A.
*AaGPRop10*	XM_001650694.1	AAEL005322	1.151	1,763,069–1,943,586	502	3	t, -	Rh7-like
*AaGPRop12*	XM_001650752.2	AAEL005373	1.153	1,048,301–1,134,510	412	5	t, -	Pteropsin
*An. gambiae*								
*AgGPRop1*	XM_003435715.1	AGAP013149	AAAB01008987	15,485,448–15,486,560	370	1	t, p	Long
*AgGPRop3*	XM_003435710.1	AGAP012985	AAAB01008987	15,549,902–15,551,014	370	1	t, p	Long
*AgGPRop4*	XM_003435711.1	AGAP012982	AAAB01008987	15,548,576–15,547,464	370	1	t, p	Long
*AgGPRop5*	XM_001238570.2	AGAP001162	AAAB01008987	15,552,322–15,553,524	378	2	t, p	Long
*AgGPRop6*	XM_322000.3	AGAP001161	AAAB01008987	15,555,427–15,556,642	372	2	t, p	Long
*AgGPRop7*	XM_312478.3	AGAP002462	AAAB01008859	9,719,568–9,718,344	374	2	t, p	Long
*AgGPRop8*	XM_001688738.1	AGAP006126	AAAB01008960	13,070,327–13,075,141	379	5	t, p	Ultraviolet
*AgGPRop9*	XM_319247.2	AGAP010089	AAAB01008980	12,661,959–12,660,706	367	3	t, p	Short
*AgGPRop10*	XM_308329.4	AGAP007548	AAAB01008807	1,869,762–1,884,337	463	3	t, p	Rh7-like
*AgGPRop11*	XM_312503.4	AGAP002443	AAAB01008859	10,064,831–10,075,172	460	5	t, -	Pteropsin
*AgGPRop12*	XM_312502.2	AGAP002444	AAAB01008859	10,056,116–10,044,185	433	5	t, p	Pteropsin
*Cx. quinquefasciatus*								
*CqGPRop1*	XM_001845645.1	CPIJ004067	3.60	303,692–304,879	374	2	-	Long
*CqGPRop2*	XM_001846589.1	CPIJ005000	3.82	144,908–146,098	378	2	-	Short
*CqGPRop3*	XM_001851105.1	CPIJ009246	3.237	371,783–376,427	379	4	t, -	Ultraviolet
*CqGPRop4*	XM_001861603.1	CPIJ011419	3.327	113,980–120,849	466	5	-	Rh7-like
*CqGPRop5*	XM_001862130.1	CPIJ012052	3.358	153,562–154,668	368	1	t, -	Long
*CqGPRop6*	XM_001862163.1	CPIJ011571	3.361	253,314–254,429	371	1	t, -	Long
*CqGPRop7*	XM_001862165.1	CPIJ011573	3.361	260,306–261,487	373	2	t, -	Long
*CqGPRop8*	XM_001862166.1	CPIJ011574	3.361	267,771–268,892	373	1	t, -	Long
*CqGPRop9*	XM_001862168.1	CPIJ011576	3.361	285,932–287,053	373	1	t, -	Long
*CqGPRop10* ^c^	XM_001863451.1	CPIJ013056	3.447	159,930–161,031	351	1	-	Long
*CqGPRop11*	XM_001863511.1	CPIJ013408	3.452	5394–6590	377	2	-	Short
*CqGPRop12*	XM_001864516.1	CPIJ014334	3.529	288,784–316,273	411	5	-	Pteropsin
*CqGPRop13*	XM_001870251.1	CPIJ020021	3.2251	5950–7074	374	1	t, -	Long

^a^Annotations from Hill et al. [25], Nene et al. [13] and Arensburger et al. [14]. Revised opsin annotations were produced using the AaegL1.2, AgamP3.5 and Cpip1.2 gene sets from VectorBase; ^b^Expression evidence for *Ae. aegypti, An. gambiae* and *Cx. quinquefasciatus* obtained from PCR or RT-PCR data (Additional file 1: Table S3), ESTs (Additional file 1: Table S2), Microarrays [40–42], RNAseq [43, 53] and mass spectrometry [44–46]; p, peptide evidence; t, transcript evidence; -, absence of transcript or peptide evidence; ^c^Indicates incomplete gene model; *N.A.* not available

Conservation was observed in the architecture between mosquito opsins. The LW opsins have no or one intron, SW opsins have one intron (except *AgGPRop9* which has two), UV opsins have four introns (except *CqGPRop3* which has three), pteropsin-like genes have four introns, and orthologs of the *D. melanogaster* Rh7 posses two introns (except *CqGPRop4* which has four) (Table 1). The *Ae. aegypti* LW opsin introns are approximately 5–6 times longer than those of *An. gambiae* and *Cx. quinquefasciatus* (Additional file 1: Table S1), consistent with the gene architecture observed in this species [13].

**Fig. 1:**
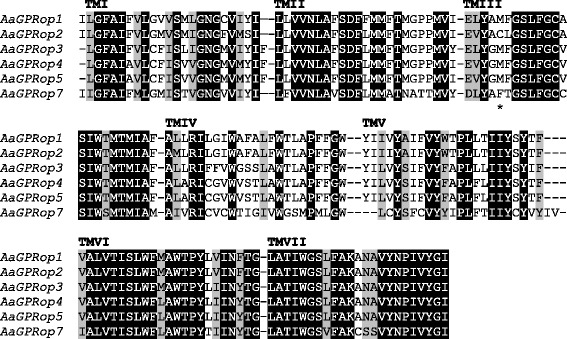
Amino acid alignment of the predicted trans-membrane domains (TMDI-VII) of *Ae. aegypti* putative LW opsins. Highlighted amino acids show positions conserved in LW opsins. *Black* shading, identical residues; *gray* shading, similar residues, based on the similarity matrix BLOSUM62. The asterisk (*) indicates the residue identified by Fitmodel as under positive selection

The putative coding sequences of the mosquito opsins were aligned to the rhodopsin from the squid *Todarodes pacificus* (TpRh) to identify amino acids and structural features, including those associated with functions in other organisms. We identified 37 and 22 residues conserved among mosquito LW opsins and Class A Rhodopsin-like GPCRs and opsin visual pigments, respectively (Additional file 1: Figure S1). Amino acids important for the interaction between the bovine rhodopsin and G-proteins are conserved in the mosquito opsins. Three classes of post-translational modifications characteristic of opsins (N-glycosylation, palmitoylation and phosphorylation) were predicted for the mosquito opsins based on comparison to the crystallized bovine rhodopsin and the squid rhodopsin [35]. These include sites in the N-terminus (N2 and N15 in bovine rhodopsin and N2 and N14 in squid rhodopsin) that undergo N-glycosylation during biosynthesis [36], sites in the C-terminus (C322 and C323 in bovine rhodopsin and C336 and C337 in squid rhodopsin) that undergo palmitoylation, the role of which in opsins remains undefined [37], and several S and T residues located in the C-termini of the bovine and squid rhodopsins that are the potential targets for phosphorylation by rhodopsin kinase [36]. The TMDs of the mosquito opsins were predicted using a hidden Markov model (HMM) using the TMHMM Server v. 2.0 [38] and aligned with Muscle. The TM domains of *Aa*GPRop1-5 and 7, *Ag*GPRop1 and 3–7 and *Cq*GPRop1, 5–9 and 11 and 13, share a minimum 62%, 59 and 68% amino acid identity (Fig. 1, Additional file 1: Figure S2A and B), respectively.

### Detection of mosquito opsin transcripts

Published EST studies support the production of transcripts for *AaGPRop1-5, 8-9_1*; *AgGPRop1-6, 8–9* and *CqGPRop3, 5–9* and *13* (Table 1, Additional file 1: Table S2). We reviewed published meta-analyses of whole body or individual organs to extract data regarding opsin temporal and spatial expression using the VectorBase Expression tool [39] (summarized in Table 1). Microarray data support the production of transcripts for all *Ae. aegypti* and *An. gambiae* opsins and *CqGPRop5*, *6* and *7* [40–42]. PCR and RT-PCR studies support transcripts for all *Ae. aegypti* opsin genes (except *AaGPRop7* and *11*) and all *An. gambiae* opsin genes (except *AgGPRop7* and *11*) (Additional file 1: Table S3, Figure S3), and RNAseq experiments support the production of transcripts for all *Ae. aegypti* and *An. gambiae* opsins [40, 43]. Peptide expression evidence was supported with mass spectrometry (LC-MS/MS) for all *An. gambiae* opsin proteins (except for AgGPRop11) [44–46]. Collectively, these public data provide evidence for expression of 28 of the 34 mosquito opsins.

Support for the production of mosquito transcripts was extended in the present study using RT-PCR analyses and includes transcripts corresponding to 10 *Ae. aegypti* and 11 *An. gambiae* opsin genes. Transcripts were detected for all *Ae. aegypti* opsin genes by RT-PCR, except for *AaGPRop4* in male and female adults and *AaGPRop4* and *10* in larvae and pupae (Additional file 1: Figure S3, Tables S3, S4). Transcripts corresponding to opsin genes were not detected in *Ae. aegypti* embryos. All PCR amplicons were sequenced to confirm coding regions. Transcripts for all *An. gambiae* opsin genes (except *AgGPRop7* and *11*) were detected by RT-PCR in male and female adults (Additional file 1: Table S3).

### Localization of mosquito opsin genes to chromosomes

Genes for the *An. gambiae* LW opsins (*AgGPRop1, 3–7*) are co-localized on the distal portion of chromosome 2R, a region of the genome associated with multiple duplicated genes. Five genes (*AgGPRop1, 3–6*) are located within a 90 kb region, and are separated from the sixth, *AgGPRop7* by 3 Mb. Localization of *Ae. aegypti* or *Cx. quinquefasciatus* opsins to chromosomes and syteny analyses were not possible due to the fragmented genome assemblies and the lack of more complete sequence and physical maps for these species.

### Phylogenetic analyses of mosquito opsins

Two initial phylogenetic analyses were performed and employed a large survey of opsin protein (193) and nucleotide (143) sequences from diverse animals (Table 1 and data not shown) and the trees were consistent with previous phylogenetic investigations of animal opsins [5, 47]. Consistent with the phylogenies of Feuda et al. [47, 48], the mosquito opsins were placed in one of five clades, namely the LW, SW, UV, Rh7-like and pteropsin clades. Mosquitoes possess one or two putative genes for each of the UV, SW, Rh7-like and pteropsin functional groups. We identified six putative LW opsins in *Ae. aegypti*, six in *An. gambiae* and eight in *Cx. quinquefasciatus,* and observed that the mosquito LW clades comprise an increased number of duplicate opsin gene lineages relative to opsin clades from insects.

**Fig. 2:**
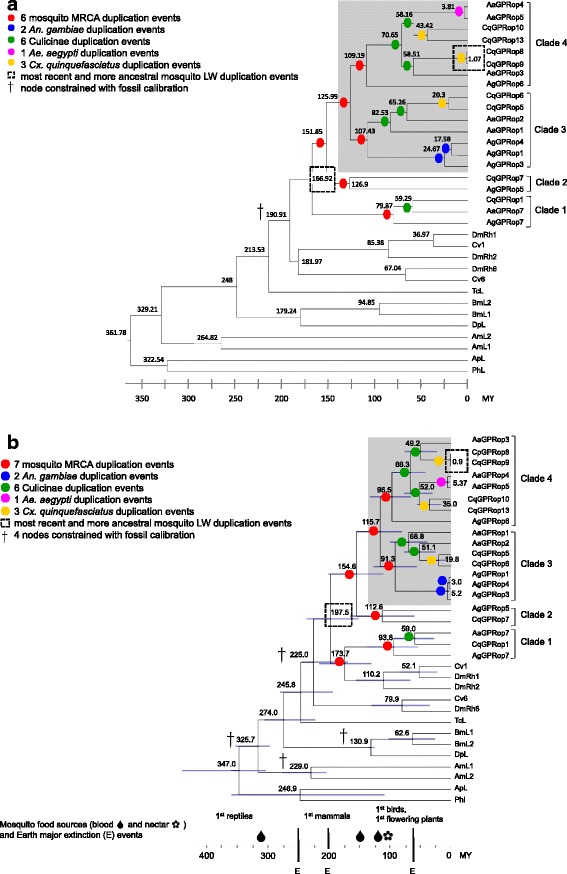
Predicted divergence times of insect long-wavelength-sensitive (LW) opsins, based on r8s and BEAST analyses. Estimates of time of divergence are shown in million years MY with r8s (**a**) and BEAST (**b**) software. *Color circles* show the duplication events in the evolution of mosquito LW opsins: most recent common ancestor (MRCA) duplication event (*red*); *An. gambiae* duplication event (*blue*); culicinae duplication event (*green*); *Ae. aegypti* duplication event (*purple*); *Cx. quinquefacitaus* duplication event (*yellow*). *Squares* (*dotted line*) show the most recent and the more ancestral mosquito LW duplication events. The *dagger* shows the constrained nodes using fossils. *Gray shading* indicates opsins under positive selection. Species abbreviations: *Acyrthosiphon pisum* (ApL), *Apis mellifera* (AmL), *Bombyx mori* (BBmL), *Calliphora vicina* (Cv), *Danaus plexippus* (DpL), *Drosophila melanogaster* (DmRh), *Pediculus humanus* (PhL), and *Tribolium castaneum* (TcL). **a** Divergence times in MY were estimated using a penalized likelihood (PL) approach and calibrated with the sister group to Culicidae (Chaoboridae, 187 MY [112]). **b** Divergence times in MY were estimated using a relaxed clock log normal model and calibrated with the following fossil calibrations [117]: *Westphalomerope maryvonneae* 313.7 MY (Holometabola), *Triassoxyela foveolata* 226.4 MY (Hymenoptera), *Parasabatinca aftimacrai* 129.4 MY (Lepidoptera), *Grauvogelia arzvilleriana* 240.5 MY (Diptera). The drops and flower represent the first predicted appearance of blood and nectar sources, namely reptiles (345–280 MY), mammals (150 MY), birds (136–65 MY) and flowering plants (125–130 MY) [120, 121]

To further investigate the retention of LW opsin genes in mosquitoes, phylogenetic analyses were conducted with 33 LW sensitive opsins from eight insect species (Additional file 1: Figure S4). The maximum likelihood tree (Additional file 1: Figure S4A) reflects the currently accepted hypothesis of insect phylogeny in which the Hymenoptera were thought to derive from an early branch of the holometabolous insects [49, 50]. The r8s and BEAST results (Fig. 2) are consistent with the estimated divergence dates (shown in parentheses) reported by Misof et al. [50], suggesting that the hemimetabolous and holometabolous insect lineages diverged approximately 347.0–361.7 MY (361.5 MY), and predict an approximate order of origin for Hymenoptera of 325.7–329.2 MY (239.5 MY), Lepidoptera of 274.0–248.0 MY (141.4 MY), Coleoptera of 245.8–213.5 MY (269.9 MY) and Diptera of 225.0–190.9 MY (157 MY).

The placement of the *D. melanogaster* DmRh1, Rh2 and Rh6 and *Calliphora vicina* Cv6 LW opsin sequences was inconsistent between trees. These sequences form a separate clade to the mosquito LW opsins in both the Parsimony and Maximum Likelihood trees, but cluster with the mosquito LW opsins in the Bayesian inference tree constructed with MrBayes. Attempts to resolve the placement of *D. melanogaster* and *C. vicina* sequences using additional amino acid and DNA sequences from other insects and arthropods with and without manual improvement of the alignment and the deletion of 3′ and 5′ sequence, were unsuccessful.

### Molecular evolution of mosquito LW opsins

The mosquito LW opsins form four clades in the ML tree (Additional file 1: Figure S4). The most basal clade (clade 1) comprises *Ag*GPRop7, *Aa*GPRop7 and *Cq*GPRop1; these orthologs have 77.0–82.0% amino acid identity. The next most derived clade (clade 2) comprises *Ag*GPRop5 and *Cq*GPRop7; these orthologs have 68.0% amino acid identity. Clades 3 and 4 are sister clades. Clade three comprises *Ag*GPRop1, op3 and op4, *Aa*GPRop1 and op2, and *Cq*GPRop5 and op6; opsin orthologs within these clades share between 83.0 and 90.0% amino acid identity, while paralogs share between 90.0 and 100.0% amino acid identity. Clade four comprises *Aa*GPRop3–5, *Ag*GPRop6, and *Cq*GPRop8–10 and op13; opsin orthologs in this clade share between 74.0 and 88.0% amino acid identity, while paralogs share between 79.0 and 100% amino acid identity. The details of these findings are presented in the following four sub-sections.

#### Identification of amino acid residues in LW opsins under positive selection

**Fig. 3:**
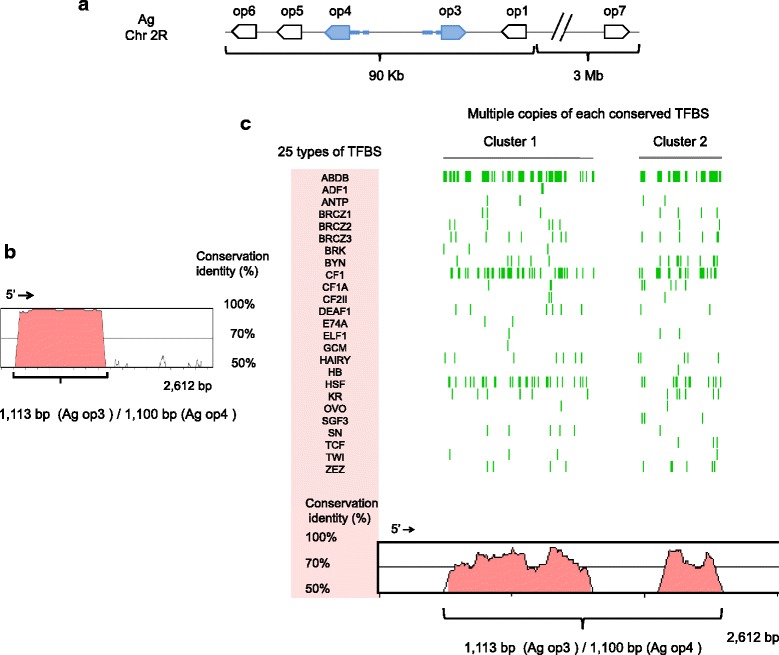
Conserved non-coding sequences (CNS) and transcription factor binding sites (TFBS) in *AgGPRop3* and *AgGPRop4*. **a** *Anopheles gambiae* opsin genes showing 5′- and 3′-non coding region on chromosome (Chr) 2R. **b** Alignment between the 5′ region of the *AgGPRop3* (2,612 bp) and of *AgGPRop4* (10,000 bp) was submitted to mVISTA using the LAGAN algorithm to identify CNS. Conserved regions are shaded (*red*). The minimum value on the Y-axis is 50%, the minimum conservation identity is 70%, and the minimum length for a CNS is 100 bp (default values on the VISTA plot). Because VISTA calculates the percentage of conservation identity as the number of matches divided by the length of the reference sequence (not the length of the alignment), the length in bp of the CNS represents the reciprocal alignment value. **c** Sequences were submitted to rVISTA to identify conserved clusters of the 50 known TFBS in insects using TRANSFAC®. Vertical (*green*) lines indicate the position of the 25 conserved TFBS identified, which occur in the clusters 1 and 2

FitModel [51] was used to test the hypothesis of adaptive changes during the evolution of duplicated LW opsins in mosquitoes (Additional file 1: Table S5). Based on the Chi-squared analysis, M0 vs M3, M3 vs M3 + S1 and M3 vs M3 + S2 were significant at *p*-value < 0.001, with M3 + S2 the best-fit model (*p*-value 0). We identified 156 (36.3%) codons showing evidence of strong negative selection and 169 codons (39.3%) under moderate negative selection. The remaining codons (105; 24.4%) were under a combination of selection pressures where for the same position within the alignment, specific residues showed evidence of strong negative selection, moderate negative selection or positive selection. Specifically, nine sites among 15 LW opsins (*Aa*GPRop1–5; *Ag*GPRop1, 3–4 and 6 and *Cq*GPRop5–6, 8–10 and 13) belonging to clades 3 and 4 (Table 2; Fig. 3) showed evidence of adaptive evolution (Table 2; Fig. 2). Five residues are located in the N-termini, three are located in the C- termini and one is located in TMDIII (Table 2).

**Table 2:** Residues in mosquito long wavelength opsins predicted under positive selection

Gene	Amino acid residue								
	N-terminus (extracellular)					Trans-membrane domain III	C-terminus (intracellular)		
Clade 4									
*AaGPRop5*	G5*	M8*	V17	A18	S19*	M128	Q357	V366	K373*
*AaGPRop4*	G5*	M8*	V17	A18	S19*	M128	Q357	V366	K373*
*CqGPRop10*	A5*	A10*	A13	V14	A15*	M124	Q340	V349	-
*CqGPRop13*	A5*	T8*	A16	A17	M18*	M127	Q356	I365	I372*
*CqGPRop8*	A5*	N8	A16	M17	V18*	M127	Q356	V365	S372
*CqGPRop9*	A5*	N8	A16	M17	V18*	M127	Q356	V365	S372
*AaGPRop3*	Q10*	Q14	A16	A17	T18*	M127	Q356	V365	S372
*AgGPRop6*	S10*	Q14	V16	V17	S18	M127	Q356	V365	A372
Clade 3									
*CqGPRop6*	D10	Q13	G15	N16	G17	C126*	D355*	S367	-
*CqGPRop5*	D10	Q13	G15	A16	G17	C126*	D355*	E364*	-
*AaGPRop2*	D10	Q13	A15	G16	G17	C126*	N357*	T366	-
*AaGPRop1*	D10	-	S14	S15	G16	M125	N358*	T367	-
*AgGPRop4*	D10	T13	S15*	G16	G17	M126	G355*	Q364	-
*AgGPRop1*	D10	T13	G15*	S16	G17	M126	G355*	Q364	-
*AgGPRop3*	D10	T13	S15*	G16	G17	M126	G355*	Q364	-

The asterisk (*) indicates residues under positive selection (Fitmodel analysis). The phylogenetic relationship of genes under positive selection is shown in Fig. 2. The residue number was derived by numbering from the first amino acid of each sequence (note that the *B. taurus* and *T. pacificus* rhodopsins were not used as reference to number the residues); -, indicates a gap inserted in the alignment and thus, a residue is not available at this position

#### Rates of evolution

Evolutionary time estimates were generated for the LW opsins using the programs r8s (Fig. 2a) and BEAST (Fig. 2b). Both analyses suggest that insect LW opsins originated from an ancestral insect opsin approximately 347.0–361.7 MY, following which paralog and ortholog genes duplicated several times, and as recently as 1.0–0.9 MY. In total, 18 (r8s) or 19 (BEAST) possible duplication events produced the complement of LW opsins in *Ae. aegypti, An. gambiae,* and *Cx. quinquefasciatus*. Six (r8s) or seven (BEAST) duplication events occurred in the most recent common ancestor (MRCA) of Culicinae and Anophelinae, representing the only section where topology differs between the trees. In r8s, the four mosquito LW opsin clades are monophyletic, and there are six events between 151.8 and 79.8 MY that occurred in the MRCA. In BEAST, the four mosquito LW opsin clades are not monophyletic; the *D. melanogaster* Rh1 and 2 and *C. vicina* Cv1 opsins form a sister clade to the mosquito clade 1 and there are two events a the base of the mosquito-fly taxa between 173.7 to 93.8 MY and four events in mosquito clades 2, 3 and 4 between 154.6 and 91.3 MY that occurred in the MRCA of Culicinae and Anophelinae. Two events are specific to *An. gambiae* and gave rise to *AgGPRop1, 3* and *4*, with predicted separation from the lineage at 5.2–24.6 MY, and subsequent divergence of *op1* and *op4* at 3.0–17.5 MY. Six events are specific to the Culicinae. r8s analyses predicted these events at 70.65 MY (*AaGPRop4* and *op5* and *CqGPRop10* and *op13,* at 58.1 MY and *CqGPRop8* and *op9* and *AaGPRop3* at 58.5 MY), 59.2 MY (*CqGPRop1* and *AaGPRop7*), 65.2 MY (*CqGPRop5* and *op6* and *AaGPRop2*), and 82.5 MY (*CqGPRop5* and *op6,* and *AaGPRop1* and *op2*). BEAST analyses predicted these events at 66.3 MY (*AaGPRop4* and *op5* and *CqGPRop10* and *op13* at 52.0 MY and *CqGPRop8* and *op9* and *AaGPRop3* at 49.2 MY), 59.0 MY (*CqGPRop1 and AaGPRop7*), 51.1 MY (*CqGPRop5* and *op6* and *AaGPRop2*), and 68.8 MY (*CqGPRop5* and *op6,* and *AaGPRop1* and *op2*). One event between 3.8 and 5.3 MY is specific to *Ae. aegypti* and gave rise to *AaGPRop4* and *op5*. Three events between 35.0–43.4, 19.8–20.3, and 0.9–1.0 MY are specific to *Cx. quinquefasciatus* and gave rise to *CqGPRop10* and *op13, op5* and *op6,* and *op8* and *op9*, respectively.

#### Intron phase

The intron phases of the mosquito LW opsins were evaluated to further explore opsin evolution (Additional file 1: Table S1) where phase “0” introns are considered more ancient than phase “1” and “2” [52]. The mosquito LW opsins are either single-exon genes or possess a single intron that is consistently in phase “0”. The mosquito pteropsin-like genes have four introns, each in different phases. The phase of the individual intron identified for the presumably more recently derived *AaGPRop3* and *AgGPRop6* is also “0”.

#### Conserved non-coding sequences (CNS) and transcription factor binding sites (TFBS)

To begin to evaluate the role of regulatory sequences in the preservation of the mosquito LW opsins, we analyzed the non-coding regions of *AaGPRop1-5, 7, AgGPRop1, 3–7* and *CqGPRop1, 5–10, 13* for putative CNS. CNS were identified in the up-stream regions of 7 of 189 LW opsin gene pair alignments (range: 1.8–48.6% nucleotide conservation between non-coding regions of gene pairs; maximum length of aligned region: 1725 bp) (Additional file 1: Table S6; Fig. 3). The *An. gambiae, Ae. aegypti* and *Cx. quinquefasciatus* genome assemblies enabled comparative analyses of conserved TFBS across these three species (Additional file 1: Table S7; Fig. 3). An average of 22.4 insect specific TFBS were identified for each of the seven gene pairs (ranging from 12 to 33 TFBS per gene pair) when all 189 possible gene pairs where aligned. The TFBS, ABDB, BRCZ2, BRCZ3, BYN, CF1, CF1A, CF2II, HSF, KR, TCF, ZEN TFBS were identified in all seven gene pairs. Two TFBS, CROC and GRH, were present in only one pair (*CqGPRop8* and *op9*). TFBS were identified only between paralog pairs and not between genes belonging to the presumably more ancestral clade 1, or between orthologous LW opsins identified to other clades. TFBS were clustered in genome regions with each cluster containing multiple binding sites for multiple transcription factors as shown in Fig. 3c for the genes *AgGPRop3* and *op4.* The lack of conservation between CNS regions and TFBS could reflect differential expression of the duplicated LW genes.

## Discussion

We describe the first detailed molecular and evolutionary analyses of the opsin gene family in the culicine mosquitoes *Ae. aegypti* and *Cx. quinquefasciatus* and the anopheline mosquito *An. gambiae.* These species exhibit different behavioral periodicities; *Ae. aegypti* is a diurnally active mosquito, while *An. gambiae* and *Cx. quinquefasciatus* exhibit nocturnal and crepuscular behaviors [18]. We report revised annotations for the published *Ae. aegypti* (10 genes; [13]), *An. gambiae* (11 genes; [25]) and *Cx. quinquefasciatus* (13 genes; [14]) opsin gene models. Transcript and amino acid sequence similarity data support the identification of functional visual and non-visual opsins in these mosquitoes*.* Transcripts were identified for each of the *Ae. aegypti* and *An. gambiae opsins* and seven of the 13 *Cx. quinquefasciatus* genes. The predicted protein sequences of the mosquito opsins contain amino acid residues conserved in other Rhodopsin-like GPCRs, including residues important for opsin function, and involved in post-translational modifications for N-linked glycosylation, palmityolation and phosphorylation.

Published microarray and RNAseq analyses provide further support for functionality of the mosquito opsins and insights into possible temporal expression. *Aedes aegypti* genes for four LW and one SW opsin (*Aa*GPRop1, 3, 4, 7 and 9) were each down regulated once at different points in a 96 h period following a blood meal, with the exception of *op3* which was down regulated twice [40]. *Aedes aegypti* RNAseq data [53] suggest two LW (*AaGPRop1* and *op4*), the UV (*op8*) and SW (*op9*) opsin genes are up regulated in sugar fed females when compared to blood feed females. These findings may help to explain why blood-fed females are largely unresponsive to external stimuli until ready to oviposit. Genes for the LW opsins *Aa*GPRop1, 2 and op5, UV (op8) and SW (op9) were up-regulated in male mosquitoes suggesting that some visual capacities may develop earlier in males than in females [40].

*In situ* hybridization studies by Hu et al. [54, 55] using anti-opsin antibodies provide evidence for expression of LW (*Aa*GPRop2), UV (*Aa*GPRop8) and SW (*Aa*GPRop9) opsins in the photoreceptor cells (PRCs) of adult *Ae. aegypti*. Mass spectrometry (LC-MS/MS) studies [44–46] provide evidence for protein expression of *An. gambiae* opsins in whole head, eye and brain. Little is currently known regarding the spectral sensitivity of the *Ae. aegypti, An. gambiae* and *Cx. quinquefasciatus* opsins. Electroretinograms (ERG) and microspectrophotometry (MSP) sensitivity studies of larval eyes (stemmata) and the adult compound eye have shown that *Ae. aegypti* exhibits two peaks of spectral sensitivity to UV (333–370 nm) and LW (500–523 nm) light [56–59], but equivalent ERG and MSP studies have not been published for *An. gambiae* or *Cx. quinquefasciatus*. Landing studies have tested the response of *Ae. aegypti* and *Cx. quinquefasciatus* to color targets, and suggest that these mosquitoes prefer red and black, and black and brown, respectively [60, 61]. Equivalent studies have not been performed for *An. gambiae*, but this species exhibited an optomotor response (*i.e*. ability to control flight speed and direction in a flight tunnel in relationship to a rotating “barber’s pole” stripe) under visible and infrared wavelengths [62].

Phylogenetic analyses placed the mosquito opsin genes into five distinct “functional” clades, namely visual UV, SW and LW opsins, non-visual pteropsins, and orthologs of the *D. melanogaster* opsin Rh7. Phylogenetic predictions for *Aa*GPRop2 (LW), op8 (UV) and op9 (SW) are supported by functional studies [55] that report dual spectral sensitivity peaks of 500–550 nm and ~350 nm (LW range) for *Aa*GPRop2, and peaks of 350 nm (UV range) for op8 and 400–450 nm (SW range) for op9. In *Apis mellifera* (honey bee) workers and drones, the non-visual pteropsin is expressed in the brain and is thought to function in regulation of circadian rhythm [63]. The wavelength sensitivity of Rh7 has not been determined and its role in *D. melanogaster* vision is not known [10]. The mosquito orthologs of Rh7 are located in a sister clade to the UV and SW clades, indicating that these receptors may respond to short wavelengths. These results corroborate other phylogenetic studies and support hypotheses for the relative divergence times of the four major holometabolus orders, with Hymenoptera as the more ancient [49] and Coleoptera, Diptera and Lepidoptera as more derived orders, as reviewed by Grimaldi and Engel [64], and supported by the recent work of Misof et al. [50]. As suggested by Cameron and Mardulyn [65], these findings highlight the utility of LW *opsins* for resolution of higher-level phylogenetic relationships.

Expansions of LW opsins have been noted in insects and the aquatic invertebrates *D. pulex,* dragonflies and stomatopods. We observed an expansion of putative LW opsins in *Ae. aegypit* (six genes)*, An. gambiae* (six genes) and *Cx. quinquefasciatus* (eight genes) relative to other insects, which typically have between one to four LW opsins. Studies suggest that the order Diptera (flies and mosquitoes) arose in the Carboniferus approximately 157 MY [50] and that the divergence of the Culicinae and Anophelinae occurred in the Permian, Triassic or Jurassic, possibly between 145 and 200 MY [15] or in the Triassic period at approximately 226 MY [16]. Our results suggest that the 20 LW opsins arose via multiple gene duplication events in the most recent common ancestor (MRCA) of the Anophelinae and Culicinae lineage in the Jurassic, approximately 197.5–166.9 MY, following which independent duplications occurred at least once in each of the three mosquito lineages. The mosquito putative LW opsins share significant amino acid identity (60–100%) but limited nucleotide conservation in the non-coding regions between paralogs. The studies of Lynch and Conery [66, 67] suggest that while the rate of origin of new gene duplicates may be high (approx. 0.01/gene/MY), the rate of duplicate preservation is low, and the authors predict the average half-life of a gene duplicate is approximately 4.0 MY [68]. The retention of duplicated LW genes in mosquitoes over long evolutionary periods implies the functional importance of their gene products.

Supporting the theory of duplication of LW opsins is the observation that five *An. gambiae* LW opsins (*AgGPRop1, 3–6*) are tandemly arrayed within a 90 kb region on chromosome 2R, and are separated from a sixth, presumably more ancestral LW opsin (*AgGPRop7*), by approximately 3 Mb. Conservation of syntenic blocks has been observed between *An. gambiae*, *Ae. aegypti,* and *Cx. quinquefasciatus* [14]. The production of improved assemblies and physical maps for the latter two species will permit studies of the *opsin* synteny among these species and may similarly illuminate gene evolution in *Ae. aegypti* and *Cx. quinquefasciatus*.

The identification of multiple putative LW sensitive opsins in the three mosquitoes could reflect an adaptation to photic environments involving LW light, as suggested by Futahashi et al., [24]. Adult *Ae. aegypti*, *An. gambiae* and *Cx. quinquefasciatus* exhibit differences in times of peak activity (*i.e.,* diurnal versus nocturnal/crepuscular) but all are active at periods when long wavelength light predominates. *Aedes aegypti* oviposition peaks at sunset [69, 70] when longer wavelengths are more abundant. Studies have shown that *Ae. aegypti* larvae and adults are capable of responding to UV and visible light, with the highest peak of sensitivity between 500 and 523 nm – *i.e*., in the LW spectrum [56–59]. Both *An. gambiae* and *Cx. quinquefasciatus* exhibit activity peaks between 22:00 and 02:00 h [71] and enter dwellings to blood feed usually when occupants are asleep [72]. Moonlight is long-wavelength-shifted and nocturnal mosquitoes are in general, more active during moonlit nights [73]. Additionally, the larvae and pupae of *Ae. aegypti, An. gambiae* and *Cx. quinquefasciatus* are active in shallow aquatic environments that are typically associated with longer wavelengths due to the defraction of incident light [74]. Supporting this theory is the identification of extreme expansions in the LW opsins in the aquatic invertebrate, *Daphnia pulex* (25 LW opsins), stomatopods (six LW opsins) [22, 23] and dragonflies (from 8 to 21 LW opsins). LW opsin expansions have also been noted in cave fish (*Astyanax fasciatus*; two or three LW opsins) and the guppy, *Poecilia reticulate* (two to six LW opsins) [75, 76]. The present study provides the first comparative genomics analyses of LW opsins in mosquitoes. RT-PCR studies reported here support expression of all opsins except *Aa*GPRop4 (LW) and op10 (Rh7-like) in *Ae. aegypti* 4th instar larvae and pupae, and a role in visual processes in immature mosquitoes. Little is known regarding opsin expression in mosquito larvae and pupae and the possible link between aquatic life-style and the duplication and retention of LW opsin deserves further investigation.

Six (r8s software) or seven (BEAST software) duplication events involving LW opsins are shared between the Culicinae and Anophelinae, suggesting retention following events at approximately 197.5–166.9 to 79.8–173.7 MY in the Mesozoic. It is possible that LW opsins evolved following a variety of events such as asteroid impacts or volcanic activity toward the end of the Mesozoic when sun- and moon-light would presumably have been reduced, or the appearance of diverse vertebrate hosts during the Cenozoic period. Based on the morphology of mosquito ommatidia, Kawada et al. [77] proposed that “crepuscular behavior of mosquitoes is a transitional behavior in the course of evolution of nocturnal behavior to diurnal behavior”, explained as a consequence of either environmental changes and/or behavioral changes in vertebrate hosts. Diversification of LW opsin function (*i.e*. spectral sensitivity) has not been established in mosquitoes, but could facilitate crepuscular and diurnal behavior. Interestingly, it has also been proposed that inversion complexes on the *An. gambiae* chromosome arm 2R (region of co-localization of LW opsin genes) are associated with ecological adaptations that increase the fitness of the carriers [78, 79]. Analyses of synteny among *An. gambiae*, *Ae. aegypti* and *Cx. quinquefasciatus* will permit further investigation of this hypothesis.

Two LW opsin duplication events are specific to the Anopheline and six to the Culicine and may reflect lineage and species-specific light detection capabilities. This hypothesis is supported by the observation of key morphological differences between the eyes of *Aedes, Anopheles,* and *Culex* species. The *Ae. aegypti* rhabdom is longer and cylindrical and the lens of the ommatidium is smaller and less hemispheric in comparison to that of *An. gambiae* [77]. Key morphological differences in the size and form of the facet lenses, rhabdom and the interommatidial angle consistently group *Ae. aegypti* with other diurnal mosquitoes, and *An. gambiae* and *Cx. quinquefasciatus* with other nocturnal mosquitoes [77, 80–82]. Thus, ommatidial structures may vary depending on the photo-environment in which mosquitoes are active and may be of limited application as a taxonomic character. Further studies are required to tease apart the contribution of LW opsins to the visual capacities of diurnal versus nocturnal mosquitoes.

The amino acid identity of the LW opsins observed both within and between mosquito taxa (approximately 70–100% between opsins and 60–100% within the TM domains) is notable considering time of divergence predictions for these duplicates. These data raise intriguing questions about the retention and conservation of LW opsin genes in the Culicinae and Anophelinae. Pseudogenization would appear unlikely and the contribution of both sub- and neofunctionalization deserves attention. In this study, we explored two possible underlying molecular mechanisms - gene selection and differential expression – that could contribute to the retention of duplicated LW *opsins* in mosquitoes.

Several studies have proposed opsin functional diversification (*i.e*., neo-functionalization) as an explanation for gene retention, and examples of this phenomenon have been described in insects. The three *D. melanogaster* LW opsins Rh1, Rh2 and Rh6, exhibit different spectral sensitivities (Rh1, 486–566 nm; Rh2, 418–506 nm; Rh6, 468–515 nm; [36]) and are expressed in different ommatidial cells and elsewhere in the body. Rh1 is the major pigment in PRCs 1–6 and is also involved in temperature discrimination by larvae [83]. Rh2 is expressed in both ocelli and testis, and Rh6 is expressed in PRC 8 and extra-retinal tissue associated with auditory processes [83]. Opsin transcripts have been identified in diverse tissues, including the rostrum, leg, abdomen, antenna, maxillary palp, proboscis and ovary of *Ae. aegypti,* and the maxillary palp and antenna of *An. gambiae* [43, 84, 85] and opsins have been identified in the antenna, maxillary palp and proboscis of *An. gambiae* [86]. Based on several lines of evidence, including work in *D. melanogaster*, Bohbot et al. [87] speculated that *Aa*GPRop1 and op2*,* expressed in the maxillary palps of adult mosquitoes, may be involved in heat sensing. The molecular role of mosquito opsins in a variety of sensory and reproductive tissues remains unclear and detailed studies are required to explore the possibility of functional diversification.

The *P. xuthus* and *P. glaucus* LW opsins, PxRh and PgRh have four amino acid substitutions in TM domain I (10Y, 23T, 29A) and TM domain III (82 F) that are predicted to “shift” the absorption spectra from green (PxRh1-2, PgRh1-2) to red (PxRh3, PgRh3) [88]. It has also been proposed that the LW genes of *D. pulex* have a role in the adaptation of the water flea to a more complex light regime in aquatic environments [22] and that the LW opsins in stomatopods diverged with respect to spectral tuning (*i.e*., amino acid substitutions, most likely in the chromophore binding pocket, that change the peak spectral sensitivity or λ_max_ value of the receptor relative to the ancestral opsin) and signal transduction (*i.e*., the receptor-mediated signaling cascade that follows interaction with a photon and produces a physiological response) [23]. The immature and adult stages of species of culicine and anopheline mosquitoes are active at low light intensities. We propose that a “suite” of opsins with different wavelength sensitivities may enable maximal capture of photons across the LW spectrum and improve visual acuity. This hypothesis is supported by the studies of Hu et al. [54, 55] who also proposed similar roles for these receptors. Physiological studies are required to explore the spectral sensitivities of mosquito LW opsins and the contributions of each opsin to light capture in mosquitoes.

Using FitModel, we identified nine residues in 15 LW opsins (*Aa*GPRop1-5, *Ag*GPRop1, 3–4, 6 and *Cq*GPRop5-6, 8–10*,* 13; Table 2) that are possibly experiencing positive selection. In preliminary analyses using PAML software (data not shown), we identified multiple candidate residues, including multiple residues in TMDIII possibly under positive selection. The modest number of residues identified only in the most recently duplicated LW opsins likely reflects the stringency of FitModel (and was the justification for selection of this software). These residues are located in the 5′ extracellular and 3′ intracellular regions and TMDIII. While further work such as site-directed mutagenesis studies are required to determine the significance of this finding, and to evaluate a possible association between TMD residues (C126 in *Cq*GPRop5, 6 and *Aa*GPRop*2*) and opsin spectral tuning, these analyses suggest that functional diversification may play a role in retention of at least some LW opsins in mosquitoes.

The prediction that the C126 residue in *Cq*GPRop5, 6 and *Aa*GPRop2 is under positive selection is significant as previous studies involving species of the Chelicerata, Crustacea and Insecta have shown that this region is important for tertiary structures (*e.g.* coil tendencies, compressibility and residue placement within the alpha-helix) associated with opsin function [89]. This region may play a crucial role in the functional diversification of arthropod opsins. Interestingly, studies in bees (family Halictidae) [90] have shown that mutations in the positively selected LW opsins may enable spectral tuning to maximize visual capabilities for foraging in dim-light in ancestral bees, thought to be diurnal. The authors identified 15 positively selected codons one of which aligns adjacent C126 in the positively selected *Aa*GPRop2, *Cq*GPRop5 and 6. This suggests a case of possible convergent evolution, as it has been proposed that mosquitoes were also diurnal feeders that developed crepuscular and night feeding behaviors.

Our discovery that nine out of a possible 430 sites may be under positive selection suggests that other mechanisms are likely responsible for the retention of the duplicated LW *opsins* over a timeframe of more than 160 MY. To evaluate adaptive changes associated with the non-coding regions of LW opsins, we examined 10 Kb of upstream sequence for the presence of conserved non-coding sequences (CNS) and transcription factor binding sites (TFBS). Binding sites for transcription factors that regulate the spatial and temporal patterns of gene expression are located in the non-coding sequence [2]. Accumulation of null mutations in the regulatory regions of recently duplicated genes that could contribute to differential gene regulation has been proposed as a mechanism for retention of duplicates [4]. Nucleotide conservation was observed in seven out of 189 alignments representing 11 LW mosquito opsins, suggesting some level of coordinated expression between at least seven paralog pairs. This also suggests the deterioration of sequence similarity across the subfamily through accumulation of mutations. The lack of DNA sequence similarity in the 5′ and 3′ non-coding regions between opsin orthologs and paralogs, and the apparent lack of common transcription units, suggests the possibility of differential opsin expression in *Ae. aegypti*, *An. gambiae* and *Cx. quinquefasciatus*. Our analysis revealed multiple copies of 25, 22, 22, 24, 34, 33, 12 and 19 different TFBS in the conserved regions between *AgGPRop3* and *op4, CqGPRop5* and *op6, AaGPRop3* and *op5, AaGPRop3* and *op5, AaGPRop4* and *op5, CqGPRop8* and *op9, AaGPRop2* and *op3, CqGPRop7* and *op8*, respectively. These TFBS are insect specific and the possibility of additional, novel TFBS in these regions can’t be ruled out. For context, shared TFBS are associated with less than 2% of genes in *D. melanogaster* (113 TFBS associated with 142 of the approximately 13,733 genes) [91].

No TFBS were identified between *AaGPRop7, AgGPRop7* and *CqGPRop1* (Clade 1, Fig. 2), supporting the hypothesis of greater time since divergence from the MRCA. Presumably, extensive accumulation of mutations in the CNS regions of these genes masks the identification of TFBS and suggests potential involvement of different regulatory units in expression. Interestingly, preliminary RT-PCR analyses detected transcripts for all opsins (except *AaGPRop4* and *op10*) in all stages and sexes of *Ae. aegypti,* and in *An. gambiae* male and female adults (except *AgGPRop7* and *11*). The studies of Rund et al. [92] suggest a more complex picture, with rhythmic expression of 12 phototransduction pathway genes in *An. gambiae* under light–dark (LD) and/or dark-dark (DD) conditions, including *AgGPRop8* (UV). Analyses of the Rund et al. [92] data identified rhythmic expression for six opsin genes under LD (*AgGPRop6*, *op8*, *op9* and *op10*) and the two pteropsins (*op11* and *12*). For the DD regime, we identified rhythmic expression for three opsin genes, two LW (*op6* and *op7*) and the Rh7 ortholog (*op10*). The studies of Dissanayake et al. [40] also support differential opsin transcript levels in *Ae. aegypti* males and females, and Hu et al. [54, 55] showed differential expression of genes for *Aa*GPRop2*,* op8 and op9 in the retina, dorsal, central, and ventral regions of the adult compound eye. Collectively, these studies suggest a complex pattern of differential gene expression in mosquitoes. Further studies are required to examine the differential expression of LW opsin genes and their possible roles in adaptive visual sensitivity across mosquito taxa.

## Conclusions

Evolutionary analyses of *Ae. aegypti, An. gambiae and Cx. quinquefasciatus* LW *opsins* suggests gene retention in the lineages Anophelinae and Culicinae, and the functional importance of these genes. Similar expansions have been observed in other aquatic invertebrates and vertebrates, with the most extreme cases in invertebrates, suggesting a gene family that is prone to duplication. Time of divergence predictions suggest mosquito opsin gene duplication events occurred in the Mesozoic and Cenozoic. Positive selection and coordinated regulation represent two mechanisms for the retention of LW opsins in the three mosquitoes. Of the 20 genes, the retention of 15 can be explained through positive selection, 11 through coordinated regulation, and 10 through both mechanisms. These genes were assigned to the more recent clades 3 and 4, with the exception of *CqGPRop7*, which is a member of clade 2. The retention of four LW opsins (*AaGPRop7, AgGPRop5* and *op7,* and *CqGPRop1*), members of the more ancestral clades 1 and 2, was not explained by either mechanism. Research is needed to resolve the spectral sensitivity, spatio-temporal expression and function of mosquito LW opsins in visual/non-visual processes. The potential connection of mosquito LW opsins to lifestyles associated with LW-dominated photo-environments (*e.g.,* fresh, shallow water) and crepuscular/nocturnal activity, deserves further investigation. Our data suggest involvement of LW opsins in lineage- and species-specific processes and provide an important foundation for future efforts directed at identifying opsin-mediated behaviors that could be exploited to achieve vector control.

## Methods

### Identification, annotation and analysis of mosquito opsins

Gene models for *Ae. aegypti, An. gambiae* and *Cx. quinquefasciatus* opsins were downloaded from VectorBase (www.vectorbase.org) [39]. Manual annotation was performed to predict the complete coding sequence by comparison to invertebrate opsin sequences and mosquito transcript data. Sequences were aligned with Multalin software [93] and annotations were produced with Artemis v7 software [94]. Gene models are listed in Table 1. The original nomenclature for the *Ae. aegypti* (*AaGPRop1-5, 7–10, 12*) and *An. gambiae* (*AgGPRop1, 3–12*) gene models proposed by Nene et al. [13] and Hill et al. [25] was retained and the *Cx. quinquefasciatus* gene models (*CqGPRop1-13*) were named for the first time in this study. The opsin protein sequences were submitted to VectorBase and comprise the official gene set for each species. Proposed gene names and putative functional annotations were also adopted by the database.

LW opsin trans-membrane domains (TMDs) were aligned with Muscle software [95] using default parameters and amino acid identity was calculated using ClustalW2 software [96]. To identify structural features and conserved amino acids, the coding sequences of the mosquito opsins were aligned with the rhodopsin sequence from the Japanese flying squid (*Todarodes pacificus*) (Protein Data Bank accession 2z73), the only invertebrate opsin for which a crystal structure is available, using Muscle software and default parameters (Additional file 1: Figure S1). Alignments were manipulated in BioEdit [97]. TMDI - VII were predicted using the TMHMM Server v. 2.0 [38]. The protein sequence, *Cq*GPRop10, which was derived from an incomplete gene model, is included in Additional file 1: Figure S2B but was excluded from the calculation of the percentage of identity.

Putative opsin post-translational modifications (glycosylation, palmitoylation and phosphorylation) were predicted using online bioinformatic tools as described below and by comparison to the squid and bovine rhodopsins [35]. Putative N-glycosylation sites were identified using NetNGlyc 1.0 (http://www.cbs.dtu.dk/services/NetCGlyc/) and palmitoylation sites were predicted with CSS-PALM 3.0 (http://csspalm.biocuckoo.org/). Putative phosphorylation sites were identified by comparison to the bovine rhodopsin sequence (multiple S and T residues, *e.g*. 12 in *D. melanogaster*, of the C-terminal region that are potential targets of rhodopsin kinase). Thirty seven amino acid residues highly conserved between Class A (Rhodopsin-like) GPCRs [98] were mapped to squid rhodopsin (shown in blue on Additional file 1: Figure S1) and used to identify equivalent residues in mosquito opsins.

Based on residues from Yokoyama and Yokoyama [99], Gartner [100], Baldwin et al. [98], Wang & Montell [36] and Arendt et al. [101], and following the nomenclature for bovine rhodopsin, the following amino acid residues were identified in mosquito opsins and mapped to squid rhodopsin (shown in orange in Additional file 1: Figure S1): K296 (the site of the Schiff base linkage to the chromophore); N2 and N15 (N-glycosylation sites); C322 and C323 (palmitoylation sites); C110 and C187 (disulfide bond sites); E113 (Schiff base counter-ion); E134 and R135 (sites important for transducing, binding and stabilizing the inactivated state of rhodopsin); W126, W265, and Y268 (sites involved in conformational changes of rhodopsin during retinal isomerization and formation of the retinal binding pocket); A117, P267, and A292 (sites affecting chromosome regeneration and activation of signal transduction); H65, H152 and H211 (sites important for conversion of metarhodopsin I to II and opsin activation-deactivation); C140 and C185 (sites involved in palmitoylation and phosphorylation); and E122 (site involved in the stabilization of metarhodopsin II). Also identified were three stretches of amino acids (E134-C140, A241-K248, N310-Q312) in the intracellular loops, IL2 and IL3, and the C-terminus considered crucial for interaction of the bovine rhodopsin with intracellular G-proteins.

### Mosquito culture

*Aedes aegypti* Liverpool strain (LVP) and *An. gambiae* SUA strain (SUA) mosquitoes were cultured at 27 °C and either 75% relative humidity (RH) (*Ae. aegypti*) or 85% RH (*An. gambiae*). Adults were maintained on a 25% sucrose solution and 11 h day: 1 h dusk: 11 h dark: 1 h dawn photoperiod. Day was simulated with both fluorescent and incandescent light. Dusk and dawn were simulated by either a gradual decrease or increase of incandescent light in the absence of fluorescent light over a 1 h period. Larvae were reared in plastic pans in RO water on ground guinea pig food (Nutriphase Products, Phoenix, AZ).

### Detection of mosquito opsin expression

#### Detection of *Ae. aegypti* and *An. gambiae* opsin transcripts by reverse transcription polymerase chain reaction (RT-PCR)

RT-PCR was used to detect opsin transcripts in four day-old sugar fed *Ae. aegypti* and *An. gambiae* male and female adults and in *Ae. aegypti* eggs (24 h post oviposition), 4^th^ instar larvae (24 h old), and pupae (24 h old). Three biological replicates each of 100 eggs, 20 larvae, 20 pupae, 50 adult male and 50 adult female *Ae. aegypti* were collected at the midpoint of the light cycle. RNA extractions were performed using TRIzol Reagent (Invitrogen, Carlsbad, CA) according to the manufacturer’s instructions. RNA was treated with RNase-Free DNAse (QIAGEN, Valencia, CA) to remove genomic DNA (gDNA). Three biological replicates of each of 25 adult male and 25 female *An. gambiae* were collected at the midpoint of the light cycle. RT-PCR was performed using the SuperScript^TM^ One-Step RT-PCR with Platinum® Taq (Invitrogen, Carlsband, CA). Where possible, primers were designed to span introns (Additional file 1: Table S8). RT-PCR was not performed for the truncated *AaGPRop*9_2 gene model. Thermocycler conditions for RT-PCR amplification of *Ae. aegypti opsin* transcripts were: 45 °C (30 min), 94 °C (3 min) followed by 35 cycles at 94 °C (30 s), 55 °C (45 s) and 72 °C (1 min), and the Lysosomal Aspartic Protease (LAP) gene was included as an internal control. Thermocycler conditions for *An. gambiae opsin* transcripts were: 45 °C (45 min), 94 °C (2 min), followed by 35 cycles at 94 °C (45 s), 56 °C (45 s), 72 °C (45 s) and finally a cycle of 72 °C (10 min), and the ribosomal protein S7 (RPS7) was used as internal control.

To further confirm opsin transcripts in *Ae. aegypti* stages and sexes, PCR was performed on cDNA synthesized from RNA of each biological replicate (egg, larvae, pupae, male and female adult) using primers described above (Additional file 1: Table S8). cDNA was synthesized using Super Script® II Reverse Transcriptase (Invitrogen, Carlsbad, CA). Genomic DNA (gDNA) was extracted from 50 adult male and 50 adult female *Ae. aegypti* (the same biological replicates as used for RNA extraction) using the Genomic-tip 100/G kit (QIAGEN, Valencia, CA). PCR cycling conditions were: 94 °C (3 min) followed by 35 cycles at 94 °C (30 s), 55 °C (45 s) and 72 °C (1 min).

All PCR products were resolved on 2% agarose gels in Tris Borate EDTA (TBE) buffer and visualized with ethidium bromide (EtBr) staining. Gel images were captured using a Gel Doc 2000 (Bio-Rad Laboratories, INC) and adjusted using Quantity One® v4.5.2 software (Bio-Rad Laboratories, INC). *Aedes aegypti opsin* amplicons were extracted form agarose gels and purified using the QIAquick Gel extraction kit (QIAGEN, Valencia, CA). Amplicons were sequenced directly using the BigDye Terminator v3.1 Cycle Sequencing Kit (Applied Biosystems, Carlsbad, CA), according to manufacturer’s instructions. Products were purified using ethanol/sodium acetate precipitation. Sequencing was conducted at the Purdue Genomics Core Facility.

#### Meta-analyses of published mosquito transcriptome and proteome studies for opsin expression

To further confirm mosquito opsin transcript and peptide expression, database and literature searches were performed using GenBank (https://www.ncbi.nlm.nih.gov/genbank/), VectorBase (http://www.vectorbase.org/), PubMed (https://www.ncbi.nlm.nih.gov/pubmed) and Web of Science (https://webofknowledge.com). Sources of opsin transcript data include EST sequences (Additional file 1: Table S2), microarray [34–36] and RNAseq [40–43, 53] data, and mass spectrometry [44–46] data for opsin peptides.

### Phylogenetic analyses of mosquito opsin

Phylogenetic analyses were conducted to determine the phylogenetic position of mosquito opsins relative to (a) opsins from other invertebrates and vertebrates, and (b) opsins from other arthropods. Opsin sequences were downloaded from GenBank (Additional file 1: Table S9). Opsins gene models for the silkmoth (*Bombyx mori*) and red flour beetle (*Tribolium castaneum*) were obtained from Velarde et al. [63]. Gene models for the waterflea (*Daphnia pulex*) were obtained from the EnsemblMetazoa database (http://metazoa.ensembl.org/Daphnia_pulex/Info/Index) and Colbourne et al. [22]. Opsin gene models for the Lyme disease tick (*Ixodes scapularis*) were obtained from Gulia-Nuss et al. [102].

Opsin phylogenies were built using (1) 193 opsin protein sequences from four animal phyla with image-forming eyes (Mollusca, Annelida, Arthropoda and Chordata) [103] and (2) 145 arthropod opsin nucleotide sequences. Sequences were aligned using the program Muscle [95]. Models of molecular evolution used to describe probabilities of amino acid or nucleotide change were used for the reconstruction of a Maximum Likelihood (ML) phylogeny. Models were estimated using ProtTest v.2.1 [104] for amino acid sequences and MrAIC v1.4.4 (https://github.com/nylander/MrAIC), [105]) for nucleotide sequences. The ML trees were constructed using RAxML 7.0.4 [106] and 1000 bootstrap replicates. Phylogenetic trees were drawn with FigTree v1.2.3 (http://tree.bio.ed.ac.uk/software/figtree/). The best-fit model of molecular evolution for the phylogeny of 193 sequences was that of LG + I + G + F, followed by LG + G + F and WAG + I + G + F, according to the Akaike Information Criterion (AIC), the corrected Akaike Information Criterion (AICc), and the Bayesian Information Criterion (BIC). The best-fit model of molecular evolution for the phylogeny of 143 sequences was that of GTR + I + G, according to AIC, AICc and BIC.

### Molecular evolution of mosquito LW opsins

A third phylogeny was constructed to examine the evolution of the mosquito putative LW opsin genes and estimate the timing of duplication events (Additional file 1: Figure S4). Maximum Likelihood, Bayesian and Parsimony trees were constructed using the coding sequences of the putative LW opsins from *Ae. aegypti* (*AaGPRop1-5, 7*)*, An. gambiae* (*AgGPRop1, 3–7*), and *Cx. quinquefasciatus* (*CqGPRop1, 5–10, 13*). Thirty-three putative LW opsins from the body louse *Pediculus humanus,* the pea aphid *Acyrthosiphon pisum*, the honey bee *Apis mellifera*, the monarch butterfly *Danaus plexippus*, the silk moth *Bombyx mori*, the red flour beetle *Tribolium castaneum*, the blowfly *Calliphora vicina*, and the fruit fly *Drosophila melanogaster* were included in the phylogeny. Nucleotide sequences were aligned with RevTrans [107]. Models of molecular evolution and trees were produced as previously described for the ML method, except that the software PhyML v3.0 [108] was used to construct the ML tree. Additionally, trees were also constructed with Parsimony and Bayesian methods, using the software PAUP v.4.0b [109] and Mr Bayes v3.1.2 [110] using 1000 bootstrap replicates and 1 million generations.

As estimated by MrAIC software, the best-fit model of molecular evolution was SYM + I + G, according to AIC, AICc and BIC. GTR, the second best model in MrAIC, was employed as SYM is not implemented in PhyML software. Other parameters of the model such as equilibrium frequencies, proportion of invariable sites and gamma distribution parameter were optimized. The number of substitution rate categories was set to 10. The “middle” of each rate class was estimated with the mean. The starting tree topology was refined with parsimony and its topology was optimized with both nearest-neighbor interchanges (NNI) and subtree pruning and regrafting (SPR) approaches. For PAUP software, the analysis was set using the sequences of the hemimetabolus insects *Ac. pisum* (ApL) and *P. humanus* (PhL) as outgroup and the ingroup was made monophyletic. The best tree of the heuristic search was saved. For MrBayes software, the evolutionary model was set to SYM + I + G and the model priors were set as follows. The substitution rates and nucleotide frequencies of the SYM model were set to flat Dririchlet (all values were 1.0) and fixed-equal, respectively. The shape parameter of the gamma distribution and the proportion of invariable sites were set to uniform with 0.1, 50.0 and 0.0, 1.0 respectively. The model was run until the standard deviation of split frequencies was below 0.01 and the potential scale reduction factor (PSRF) was close to 1.0. The parameter values and the trees were summarized discarding the first 25% of the samples.

FitModel v3.2.17 was used (http://compevol.wordpress.fos.auckland.ac.nz/category/software/, [51]) to identify residues under positive selection in mosquito putative LW opsin genes. FitModel calculates the synonymous/synonymous rate ratio (d_N_/d_S_ = ω) and permits substitutions between codons under three selection regimes, namely (1) a negative (purifying) regime (ω1), (2) a strictly or nearly neutral regime (ω2), and (3) a positive selection regime (ω3). Four models were considered: M0, M3, M3 + S1 and M3 + S2. M0 considers all sites as under the same selection process (*i.e.* ω constant at all sites). M3 considers selective constraint across sites for three rate ratio classes, ω1 < ω2 < ω3, with selection classes corresponding to either strong purifying selection (0 < ω1 < < 1), weak purifying or diversifying selection (ω2 ≃ 1) or strong positive selection (ω3 > 1). FitModel allows for a combined substitution and switching process (S, changes between switches or selection classes). M3 + S1 and M3 + S2 employ M3 and consider equal switching rates among selection categories (S1) (*i.e*. ω1 = ω2 = ω3) and unequal rates of switching among ω1, ω2 and ω3 selection categories (S2), respectively. The log likelihood of the models was compared using a Chi-squared test. For the best model, the non-synonymous/synonymous nucleotide substitution rate ratio (d_N_/d_S_ or ω) was analyzed to determine if there was evidence of selection.

To estimate the divergence times (with confidence intervals) of mosquito LW opsins the following steps were performed with r8s and BEAST software. The ML tree from PhyML analyses with branch lengths estimated assuming a GTR + I + G model of molecular evolution was used to estimate divergence times using r8s v1.71 software [111]. r8s uses variation in the substitution rates along branches of the phylogeny in a statistical framework. The calibration was set at 187 ± 1 million years based on the fossil evidence for Chaoboridae, the sister clade to Culicidae [112]. We used the penalized likelihood (PL) approach, a semi-parametric smoothing method [113], to estimate nucleotide substitution rates and ages, allowing evolutionary rates to vary smoothly between branches. To find an optimum rate smoothing parameter, the truncated Newton (TN) optimization algorithm was used [113].

Using MEGA 7 [114], a molecular clock test was performed by comparing the maximum likelihood value for the given topology with and without the molecular clock constraints under Tamura-Nei model [115]. All positions containing gaps were eliminated. The null hypothesis of equal evolutionary rate through the tree was rejected at a 5% significance level (*p* = 0). To facilitate establishment of the evolutionary model and selection of options for the Markov chain Monte Carlo (MCMC) analysis, BEAUti 2.4.4 was used [116]. This software converted the aligned sequences of 33 LW opsins from NEXUS format into BEAST XML format (the XML is provided as Additional file 2). BEAST simultaneously estimates divergence-time and phylogenetic relationships. The substitution model was set based on MrAIC previous results, GTR model plus gamma (10 categories), plus invariant sites (0); the shape parameter and substitution model were set to be estimated; the frequencies were set to empirical. The clock model was chosen based on MEGA 7 results, the clock model was set to ‘relaxed clock log normal’. The ‘Calibrated Yule model’ was used as the tree prior. The absolute estimates of divergence times were calculated from the following fossils calibrations [117]: *Westphalomerope maryvonneae* 313.7 MY (Holometabola), *Triassoxyela foveolata* 226.4 MY (Hymenoptera), *Parasabatinca aftimacrai* 129.4 MY (Lepidoptera), *Grauvogelia arzvilleriana* 240.5 MY (Diptera), monophily was enforced in these four nodes. For the MCMC options a chain length of 20,000,000 was set with a log sample every 1000 steps. BEAST 2.3.2 [116] was run using the XML input file and the output, a log file, was analyzed with Tracer v1.6.0 software [116] to produce a graphical and quantitative summary of results. To summarize BEAST posterior sample of phylogenetic time-trees along with its sample parameter estimates, the program TreeAnnotator v2.4.4 [116] was used. Based on the specified chain length and frequency of the sampling steps, the trees file contains 20,000 trees, and to specify a 1% burn in the value 200 was used. The posterior probability limit was set to zero to annotate all nodes. Visualization of node values and confidence intervals, and cosmetic editions to the tree were performed with FigTree 1.4.3 (http://tree.bio.ed.ac.uk/software/figtree/).

### Opsin intron phase analysis

Opsin splice junctions were analyzed to identify genes with more derived or more ancient intron phases. The intron phase was designated as “0” when positioned between two codons, phase “1” when the intron disrupted a codon after the first base position, and phase “2” when the intron disrupted the codon after the second base.

### Identification of conserved non-coding sequences (CNS) and transcription factor binding sites (TFBS)

The typical CNS may be several thousand base pairs in length and may comprise multiple transcription factor binding sites (TFBS) or enhancers that can send information to the core promoter of a gene. New or mutated TFBS are of interest as they can turn on or off transcription and contribute to differential gene regulation. VISTA software (http://genome.lbl.gov/vista, [118, 119]) was used to predict CNS and putative TFBS in the 5′ non-coding regions of the *Ae. aegypti, An. gambiae* and *Cx. quinquefasciatus* putative LW opsins. Global pair-wise alignments of up to 10 kb of 5′ non-coding sequence from the 20 mosquito LW opsins were produced with mVista software [118]. The CNS VISTA curve identifies conserved regions using the LAGAN algorithm. Results are shown in a graph (VISTA plot) with the following characteristics: the minimum value on the y-axis = 50%, the minimum conservation identity = 70%, and the minimum length for a CNS = 100 bp. rVISTA [119] was used to identify putative TFBS using TRANSFAC® 50 insect matrices and a comparative sequence analysis. rVISTA predictions of conserved binding sites are defined as sites located in the sequence fragments conserved between two species at greater than 80% identity over a 24 bp window.

The presence of genes located up-stream of LW mosquito opsins limited the CNS and TFBS analysis. The 20 sequences where aligned against each other producing a total of 189 alignments. The available length of the 5′ (up-stream) regions used in analyses is as follows: *AaGPRop1*, 10,000 bp; *AaGPRop2*, 10,000 bp; AaGPRop3*, 10,344 bp; *AaGPRop4*, 10,000 bp; *AaGPRop5*, 1,307 bp; *AaGPRop7*, 10,000 bp; *AgGPRop1*, 799 bp; AgGPRop3*, 2,612 bp; AgGPRop4*, 10,000 bp; AgGPRop5*, 1,307 bp; AgGPRop6*, 1,902 bp; *AgGPRop7*, 10,000 bp; *CqGPRop1*, 10,000 bp; *CqGPRop5*, 5,949 bp; *CqGPRop6*, 10,000 bp; CqGPRop7*, 5,734 bp; CqGPRop8*, 6,283 bp; CqGPRop9*, 3,546 bp; *CqGPRop10*, 10,000 bp; *CqGPRop13*, 5,949 bp. The asterisk (*) designates those genes for which the 5′ region is also the 3′ (down-stream) region of a neighboring opsin gene.

## Additional files

Additional file 1: Suplementary tables and figures. (DOCX 925 kb)
Additional file 2: BEAST file in XML format. (XML 68.8 kb)

## Abbreviations

AIC: Akaike Information Criterion
AICc: Corrected Akaike Information Criterion
BIC: Bayesian Information Criterion
CNS: Conserved Non-coding Sequences
DD: Dark-dark
EL: Extracellular loops
GPCR: G protein-coupled receptor
IL: Intracellular loops
LD: Light-dark
LW: Long-wavelength
MCMC: Markov chain Monte Carlo
ML: Maximum likelihood
MRCA: Most recent common ancestor
MY: Million years ago
NNI: Nearest-neighbor interchanges
SPR: Subtree pruning and regrafting
SW: Short-wavelength
TFBS: Transcription factor binding sites
TMD: Trans-membrane domain
TN: Truncated Newton
UV: Ultraviolet
