# Supplementary material for: Retention of duplicated long-wavelength opsins in mosquito lineages by positive selection and differential expression
Source: BMC Evol Biol. 2017 Mar 21;17:84. doi: 10.1186/s12862-017-0910-6 (PMC5359912; doi:10.1186/s12862-017-0910-6)
Supplement: Additional file 1: — Suplementary tables and figures. (DOCX 925 kb) [file 12862_2017_910_MOESM1_ESM.docx]

**Additional File 1**

**Table S1. Predicted intron phase and intron length of *Ae. aegypti, An. gambiae and Cx. quinquefasciatus* opsins.**

|  | **Intron 1** | **Intron 2** | **Intron 3** | **Intron 4** |
| --- | --- | --- | --- | --- |
| ***Ae. aegypti* (average intron length 24,936.3 bp)** | | | | |
| *AaGPRop9_1* | 2 (12,611 bp) | - | - | - |
| *AGPRop10* | 2 (178,945 bp) | 1 (64 bp) | - | - |
| *AaGPRop12* | 0 (7,931 bp) | 2 (33,958 bp) | 1 (28,685 bp) | 0 (14,397 bp) |
| *AaGPRop3*^a^ | 0 (72 bp) | - | - | - |
| *AaGPRop7*^a^ | 0 (57 bp) | - | - | - |
| *AaGPRop8* | 2 (69 bp) | 1 (2, 450 bp) | 2 (674 bp) | 1 (44,260 bp) |
| ***An. gambiae* (average intron length 1,940.21 bp)** | | | | |
| *AgGPRop10* | 2 (13,095 bp) | 1 (89 bp) | - | - |
| *AgGPRop7*^a^ | 0 (100 bp) | - | - | - |
| *AgGPRop12* | 0 (577 bp) | 1 (3,751 bp) | 2 (6,182 bp) | 0 (120 bp) |
| *AgGPRop11* | 0 (1,367 bp) | 1 (5,144 bp) | 2 (2,268 bp) | 0 (180 bp) |
| *AgGPRop8* | 2 (78 bp) | 1 (76 bp) | 2 (75 bp) | 1 (3,446 bp) |
| *AgGPRop9* | 2 (72 bp) | 2 (81 bp) | - | - |
| *AgGPRop5*^a^ | 0 (66 bp) | - | - | - |
| *AgGPRop6*^a^ | 0 (97 bp) | - | - | - |
| ***Cx. quinquefasciatus* (average intron length 2,219.25 bp)** | | | | |
| *CqGPRop1*^a^ | 0 (63 bp) | - | - | - |
| *CqGPRop2* | 2 (54 bp) | - | - | - |
| *CqGPRop3* | 2 (56 bp) | 2 (64 bp) | 1 (3,385 bp) |  |
| *CqGPRop4* | 2 (768 bp) | 0 (1,551 bp) | 2 (3,082 bp) | 1 (62 bp) |
| *CqGPRop7*^a^ | 0 (60 bp) | - | - | - |
| *CqGPRop11* | 2 (63 bp) | - | - | - |
| *CqGPRop12* | 0 (4,412 bp) | 1 (13,925 bp) | 2 (7,824 bp) | 0 (93 bp) |

^a^ LW opsins

Note : Intron phase is shown as “0”, when positioned between two codons; “1”, when the intron disrupted a codon after the first base position; “2”, when the intron disrupted the codon after the second base. Intron length is shown in bp in parenthesis.

**Table S2. Evidence for *Ae. aegypti, An. gambiae* and *Cx. quinquefasciatus* opsin transcripts.**

| **Gene** | **Expressed Sequence Tag (EST)**  **GenBank Accession Number** | **% EST coverage of opsin coding region** |
| --- | --- | --- |
| ***Ae. aegypti*** | | |
| *AaGPRop1* | DV251445.1, DV244106.1, DV251444.1 | 100 |
| *AaGPRop2* | DV254186.1, DV243151.1 | 100 |
| *AaGPRop3* | DW199624.1, DW199064.1, DW992610.1 | 98.6 |
| *AaGPRop4* | EG004786.1 | 47.4 |
| *AaGPRop5* | DW191491.1, DW201934.1 | 75.7 |
| *AaGPRop7* | - | - |
| *AaGPRop8* | EG003561.1, DV316083.1 | 100 |
| *AaGPRop9_1* | DV413960.1, DV354251.1 | 50.0 |
| *AaGPRop9_2* | - | - |
| *AaGPRop10* | - | - |
| *AaGPRop12* | - | - |
| ***An. gambiae*** | | |
| *AgGPRop*1 | BX616920.1, CR528011.1 | 100 |
| *AgGPRop3* | CR528011.1 | 67.8 |
| *AgGPRop4* | CX818544.1, CR528011.1 | 100 |
| *AgGPRop5* | BX617017.1, CR533847.1 | 100 |
| *AgGPRop6* | BM650684.1, CR526592.1 | 100 |
| *AgGPRop7* | - | - |
| *AgGPRop8* | BM585863.1, CR531766.1 | 100 |
| *AgGPRop9* | BX623599.1, CR527268.1 | 91.5 |
| *AgGPRop10* | - | - |
| *AgGPRop11* | - | - |
| *AgGPRop12* | - | - |
| ***Cx. quinquefascaitus*** | | |
| *CqGPRop1* | - | - |
| *CqGPRop*2 | - | - |
| *CqGPRop3* | FF276264.1 | 47.4 |
| *CqGPRop4* | - | - |
| *CqGPRop5* | EV323979.1, EV312642.1 | 100 |
| *CqGPRop6* | FF251813.1, FF184231.1 | 100 |
| *CqGPRop7* | FF184232.1, EV342508.1 | 77.4 |
| *CqGPRop8* | FF184232.1 | 69.0 |
| *CqGPRop9* | FF184232. | 69.0 |
| *CqGPRop10* | - | - |
| *CqGPRop11* | - | - |
| *CqGPRop12* | - | - |
| *CqGPRop13* | FF163301.1, FF192295.1 | 87.0 |

-, No EST identified

**Table S3. Detection of mosquito opsin transcripts by RT-PCR.**

| **Gene** | **Egg** | **4^th^ instar Larva** | **Pupa** | **Adult male** | **Adult**  **female** |
| --- | --- | --- | --- | --- | --- |
| ***Ae. aegypti*** |  |  |  |  |  |
| *AaGPRop1* | **---** | **+++** | **+++** | **+++** | **+++** |
| *AaGPRop2* | **---** | **+++** | **+++** | **+++** | **+++** |
| *AaGPRop3* | **---** | **+++** | **+++** | **+++** | **+++** |
| *AaGPRop4* | **---** | **---** | **---** | **---** | **---** |
| *AaGPRop5* | **---** | **+++** | **+++** | **+++** | **+++** |
| *AaGPRop7* | **---** | **+++** | **+++** | **+++** | **+++** |
| *AaGPRop8* | **---** | **+++** | **+++** | **+++** | **+++** |
| *AaGPRop9* | **---** | **+++** | **+++** | **+++** | **+++** |
| *AaGPRop10* | **---** | **---** | **---** | **++-** | **+++** |
| *AaGPRop12* | **---** | **+++** | **+++** | **+++** | **+++** |
| ***An. gambiae*** |  |  |  |  |  |
| *AgGPRop1* | N.P. | N.P. | N.P. | +++ | +++ |
| *AgGPRop3* | N.P. | N.P. | N.P. | +++ | +++ |
| *AgGPRop4* | N.P. | N.P. | N.P. | +++ | +++ |
| *AgGPRop5* | N.P. | N.P. | N.P. | +++ | +++ |
| *AgGPRop6* | N.P. | N.P. | N.P. | +++ | +++ |
| *AgGPRop7* | N.P. | N.P. | N.P. | --- | --- |
| *AgGPRop8* | N.P. | N.P. | N.P. | +++ | +++ |
| *AgGPRop9* | N.P. | N.P. | N.P. | +++ | +++ |
| *AgGPRop10* | N.P. | N.P. | N.P. | +++ | +++ |
| *AgGPRop11* | N.P. | N.P. | N.P. | --- | --- |
| *AgGPRop12* | N.P. | N.P. | N.P. | +++ | +++ |

Note: Opsin PCR amplicon present (+) or absent (-), in one of three biological replicates. N.P., not performed.

**Table S4. Primers used to detect *AaGPRop4*, *AgGPRop7* and *AgGPRop11* transcripts.**

| **Gene** | **Primers** | **Binding regions** |
| --- | --- | --- |
|  |  |  |
| ***Ae. aegypti*** | | |
| ***AaGPRop4*** |  |  |
|  | F1: 5’-AGAAACGGAACCGGTGCCG-3’ | 5’UTR op4 |
|  | F2: 5’-TGGAGCTGACACCGGTGACAA-3’ | 5’UTR op4 |
|  | F3: 5’-ATTCTAATTGGATTTCATTTGCCG-3’ | 5’UTR op4 |
|  | F4: 5’-CATCTACTCGTACACCTTCATC-3’ | E1 op4 and op5 |
|  | F5: 5’-ATCTTCAAGGCGTCTCCAATC-3’ | E1 op4 |
|  | R1: 5’-ATTGGAGGTTGTTTGAAGGTGG-3’ | 3’UTR op4 |
|  | R2: 5’-GAATTAGATACCTACGTCCCC-3’ | 3’ UTR op4 |
|  | R3: 5’-AACATTATCAAGTGCCTTAAGCAG-3’ | 3’ UTR and E1 op4 |
|  | R4: 5’-GGTCGAACGCAATCATAGTCATG-3’ | E1 op4 and op5 |
|  | R5: 5’-ATATCTGCCGGAACTCTATCCACC-3’ | E1 op4 and op5 |
| ***An. gambiae*** | | |
| ***AgGPRop7*** |  |  |
|  | F1: 5’-TTCGGCTGCTGCTCGATC-3’ | E1 |
|  | R1: 5’-GTGGAACCAATCGTCGGTC-3’ | E1 |
| ***AgGPRop11*** |  |  |
|  | F1: 5’-CAACAACAGCAACCGGGAC-3’ | E5 |
|  | R1 : 5’-CCTACTACTACTACCACCAC-3’ | E5 |

Note : *Ae. aegypti* primers were used in all 21 possible combinations.

**Table S5. Positive selection analysis of 33 insect long wavelength opsins performed with Fitmodel software.**

|  | **M0**  **(all sites under the same selection, ω is constant)** | **M3**  **(site variation,**  **ω_1_< ω_2_<ω_3_)** | **M3 + S1**  **(site-specific shifts,**  **ω_1_=ω_2_=ω_3_)** | **M3 + S2**  **(Unequal switching rates, among ω_1_, ω_2_ and ω_3_)** |
| --- | --- | --- | --- | --- |
| **Log likelihood** | -22,582.89 | -21,608.83 | -21,443.77 | -21,263.27* |
| **ω_1_, ω_2_, ω_3_** | 0.09 | 0.01, 0.11, 0.39 | 0.00, 0.14, 0.71 | 0.00, 0.05, 19.99 |
| ***p_1_*, *p_2_*, *p_3_*** | 1.00 | 0.42, 0.39, 0.18 | 0.59, 0.28, 0.12 | 0.51, 0.47, 0.01 |
| **R_01_, R_02_, R_12_** | - | - | 1.81, 1.81, 1.81 | 0.26, 0.53, 69.34 |

S, switching model; ω, substitution rate ratios; *p*, equilibrium frequencies; R, switching rates for amino acid residues in the three rate ratio classes parameters; *, statistically significant (p < 0.001) based on Chi-square analysis of the log likelihood differences between the models; -, parameter not calculated under this model.

**Table S6. Identification of CNS and putative TFBS in the 5’ UTR of mosquito LW opsins.**

| **Length of aligned 5’ UTR (bp)** | | **CNS** | | **TFBS** | |
| --- | --- | --- | --- | --- | --- |
|  |  | Length of nucleotide conservation (bp)^†^ | Percentage of nucleotide conservation | No. of common TFBS | Number of TFBS clusters^‡^ |
| *AgGPRop3*  2,612 | *AgGPRop4*  10,000 | 1,113/1,100 | 42.6/11.0 | 25 | 2 |
| *CqGPRop5*  10,000 | *CqGPRop6*  10,000 | 397/397 | 4.0/39.7 | 22 | 3 |
| *AaGPRop3*  10,344 | *AaGPRop5*  10,000 | 227/293 | 2.2/2.9 | 22 | 3 |
| *AaGPRop4*  10,000 | *AaGPRop5*  10,000 | 267/273 | 2.7/2.7 | 24 | 1 |
| *CqGPRop8*  6,283 | *CqGPRop9*  3,546 | 1704/1725 | 27.1/48.6 | 34/33 | 2/3 |
| *AaGPRop2*  10,000 | *AaGPRop3*  10,344 | 377/379 | 3.8/3.7 | 12 | 2 |
| *CqGPRop7*  5,734 | *CqGPRop8*  6,283 | 115/115 | 2.0/1.8 | 19 | 2 |

Note: Alignment of the mosquito long wavelength **(**LW) opsins produced a total of 189 alignments. Transcription factor binding sites **(**TFBS) where identified in the 5’ UTR of seven gene pairs. VISTA calculates the conservation identity as the number of matches divided by the length of the reference sequence (not the length of the alignment). Therefore, length and percentage of the conserved non-coding sequences **(**CNS) represents reciprocal alignment values.

^†^ Minimum conservation identity is 70%.

^‡^ Types of TFBS conserved in aligned 5’- UTR region between gene pairs.

**Table S7. List of putative TFBS predicted in 5’ CNS regions of mosquitoes LW opsins.**

| **TFBS** | ***AgGPRop*** | ***CqGPRop*** | ***AaGPRop*** | ***AaGPRop*** | ***CqGPRop*** | ***AaGPRop*** | ***CqGPRop*** |
| --- | --- | --- | --- | --- | --- | --- | --- |
|  | ***3* and *4*** | ***5* and *6*** | ***3* and *5*** | ***4* and *5*** | ***8* and *9*** | ***2* and *3*** | ***7* and *8*** |
| **ABD-B** | + | + | + | + | + | + | + |
| **ADF1** | + | - | - | - | + | - | - |
| **ANTP** | + | + | + | + | + | - | + |
| **BCD** | - | - | + | + | + | - | - |
| **BRCZ1** | + | + | + | + | + | - | + |
| **BRCZ2** | + | + | + | + | + | + | + |
| **BRCZ3** | + | + | + | + | + | + | + |
| **BRCZ4** | - | - | - | - | + | - | + |
| **BRK** | + | - | - | - | + | - | - |
| **BYN** | + | + | + | + | + | + | + |
| **CF1** | + | + | + | + | + | + | + |
| **CF1A** | + | + | + | + | + | + | + |
| **CF2II** | + | + | + | + | + | + | + |
| **CROC** | - | - | - | - | + | - | - |
| **DEAF1** | + | + | + | + | + | - | - |
| **DL** | - | + | + | + | + | - | + |
| **DREF** | - | - | + | - | + | - | + |
| **E74A** | + | - | - | - | + | - | - |
| **ELF1** | + | + | + | + | + | - | - |
| **EN** | - | + | - | + | + | - | - |
| **GCM** | + | - | - | - | + | - | - |
| **GRH** | - | - | - | - | + | - | - |
| **HAIRY** | + | + | + | + | + | - | - |
| **HB** | + | + | + | + | + | - | + |
| **KR** | + | + | + | + | + | + | + |
| **MTTFA** | - | - | - | + | + | - | + |
| **OVO** | + | + | - | - | + | - | - |
| **SGF3** | + | + | - | + | + | - | - |
| **SN** | + | - | + | + | + | + | - |
| **TCF** | + | + | + | + | + | + | + |
| **TWI** | + | + | + | + | + | - | + |
| **ZEN** | + | + | + | + | + | + | + |
| **ZESTE** | - | - | - | - | +/- | - | - |

ABD-B, Abdominal B; ADF1, Adh transcription factor 1; ANTP, Antennapedia; BCD, Bicoid; BRCZ1-4, broad; BRK, Brinker; BYN, Brachyenteron; CF1, ultraspiracle; CF1A, Ventral venis lacking; CF2II, Chorion factor 2 isoform II; CROC, Crocodile; DEAF1, Deformed epidermal autoregulatory factor-1; DL, (no information available); DREF, DNA replication-related element factor; E74A, Ecdysone-induced protein 74EF; ELF1, Grainy head; EN, engrailed; GCM, glial cells missing; GRH, grainy head; HAIRY, hairy; HB, hunchback; HSF, Heat shock factor; KR, Kruppel; MTFA, Mitochondrial transcription factor A; OVO, ovo; SGF3, POU domain protein; SN, singed; TCF, Pan pangolin; TWI, Twist; ZEN, zerknullt; ZESTE, Suppressor of zeste 3. Details regarding transcription factor binding sites (TFBS), including consensus sequence, are available from the NCBI Gene database (<http://www.ncbi.nlm.nih.gov/gene>, [1]) and TRANSFAC® (<http://www.biobase-international.com/product/transcription-factor-binding-sites>, [2]).

**Table S8. Summary of primers used to amplify *Ae. aegypti* (*AaGPRop*) and *An. gambiae* (*AgGPRop*) opsins.**

| **Gene** | **Primers** | **Binding regions** | **Expected amplicon size (bp)** | |
| --- | --- | --- | --- | --- |
|  |  |  | **cDNA** | **gDNA** |
| ***Ae. aegypti*** |  |  |  |  |
| ***AaGPRop1*** | F: 5’ GCAAGCAACACTTTACGGCTTTG 3’  R: 5’ GGTAGTCAGGTAATCGGTACC 3’ | 5’UTR and E1 | 729 | 788 |
| ***AaGPRop2*** | F: 5’ GCTTACCGCCAAAAGTTATCAGC 3’  R: 5’ GTACGTTCTGTAGCGCATTTAGG 3’ | 5’UTR and E1 | 1,238 | 1,302 |
| ***AaGPRop3*** | F: 5’ CTTCGCTCGCCTGGACACTC 3’  R: 5’ TTAGGCTGAGGGCTTCTCTTCTTG 3’ | E1 and E2 | 599 | 671 |
| ***AaGPRop5*** | F: 5’ GTACTTCGCGCCTCTGTTTC 3’  R: 5’ CATTATCATGTCCCTTTAAGCAG 3’ | E1,  3’UTR and E1 | 484 | 484 |
| ***AaGPRop7*** | F: 5’ GGAAGTATGCCTATGCTGGGTTG 3’  R: 5’ AGCGGCCGTTGTCTCCATCC 3’ | E1 and E2 | 606 | 663 |
| ***AaGPRop8*** | F: 5’ ATGCCTTTCGAAGAGCATCTGAG 3’  R: 5’ CATTGGATGGTGTTCGCAACG 3’ | E1 | 274 | 343 |
| ***AaGPRop9_1*** | F: 5’ CGGCGCCCCCATATTATATG 3’  R: 5’ AGCTGGTCTCGTATCCAACC 3’ | E1 and E2 | 237 | 12,848 |
| ***AaGPRop10*** | F: 5’ GGTCATCAATCTGGCGATCG 3’  R: 5’ TGCATGCGGCCGTCTTAGC 3’ | 5’UTR and E1 | 674 | 738 |
| ***AaGPRop12*** | F: 5’ CAAACGCTGAATGCCACCTC 3’  R: 5’ CGAAACTGCACTCCACGATTTC 3’ | E2 and E3 | 457 | 14,854 |
| **LAP** | F: 5’ CTCGTTTGCTTGGCCGTTC 3’  R: 5’ TCGTAACGGAAACCCCTCC 3’ | E1 and E4 | 437 | 757 |
| ***An. gambiae*** |  |  |  |  |
| ***AgGPRop1*** | F: 5’ ATGCCTGGACGCAGGGATC 3’  R: 5’ AACGGTCAAAGGCAATCATC 3’ | E1 | 287 | N.P. |
| ***AgGPRop3*** | F: 5’ TCATCTACGCCATCTTCGTG 3’  R: 5’ ATTTCATTGCCTTGCTTGCT 3’ | E1 and 3’UTR | 532 | N.P. |
| ***AgGPRop4*** | F: 5’ CGCTGCTGACCATCATCTAC 3’  R: 5’ TTTGCATCTCGTTCGATTGT 3’ | E1 and 3’UTR | 554 | N.P. |
| ***AgGPRop5*** | F: 5’ CGGTCATCGGATGCGTCC 3’  R: 5’ CGTTCTCCTTACGCAGTTG 3’ | E2 | 280 | N.P. |
| ***AgGPRop6*** | F: 5’ TTCGCAGAGCCGCACTTC 3’  R: 5’ ACGAAGCCAGAGTAGATCAG | E1 | 653 | N.P. |
| ***AgGPRop8*** | F: 5’ CTACCTGTCTGGCACGTTC  R: 5’ CGACAAACTTGCAGGCACAG 3’ | E5 | 362 | N.P. |
| ***AgGPRop9*** | F: 5’ TCTCGGAGCGACTGGTCG 3’  R: 5’ ACGGACGTGACCGAAGAG 3’ | E2 | 392 | N.P. |
| ***AgGPRop10*** | F: 5’ TCGAGCAAGAGCAAGAACA 3’  R: 5’ GACTCTTCACCACCACCAC 3’ | E3 | 353 | N.P. |
| ***AgGPRop12*** | F: 5’ CGAGCTGACCAAGAGCAG 3’  R: 5’ AAGCGTGTACCGGCATAAG 3’ | E5 | 255 | N.P. |
| **RPS7** | F: 5’ GGCGATCATCATCTACGT 3’  R: 5’ GTAGCTGCTGCAAACTTCGG 3’ | E1 and 3’UTR | 450 | N.P. |

LAP, lysosomal aspartic protease positive control; RPS7, ribosomal protein S7; E, exon; UTR, untranslated region; N.P., not performed.

**Table S9. Accession numbers of opsin sequences used to construct phylogenetic trees.**

| **Phylum, Subphylum, Species (Species Abbreviation)** | **Genbank accession number** |
| --- | --- |
| **Chordata, Cephalochordata** |  |
| *Branchiostoma belcheri* (Bb) | AB050606.1 (Bb1), AB050607.1 (Bb2), AB050610.1 (Bb3), AB050608.1 (Bb4), AB050609.1 (Bb5), AB050611.1 (Bb6) |
| **Chordata, Craniata** |  |
| *Geotria australis* (Ga) | AY366495.1 (GaUV), AY366492.1 (GaB), AY366494.1 (GaRhB), AY366493.1 (GaRhA), AY366491 (GaLr) |
| *Callorhinchus milii* (Cm) | EF565167.1 (CmRh1), EF565168.1 (CmRh2), EF565165.1 (CmL1), EF565166.1 (CmL2) |
| *Carassius auratus* (Ca) | L11863.1 (CaRh), L11865 (CaL1g), L11866.1 (CaL2g), D85863.1 (CaUV), L11864.1 (CaB), L11867.1 (CaL3r) |
| *Xenopus laevis* (Xl) | L07770.1 (XlRh), U23463.1 (XlV), U90895.1 (XlLr), BC169653.1 (XlM) |
| *Anolis carolinensis* (Ac) | L31503.1 (AcRh), AF134189.1 (AcRh2), AF134192.1 (AcS1), AF133907.1 (AcS2), U08131.1 (AcLr) |
| *Gallus gallus* (Gg) | D00702.1 (GgRh), M92038.1 (GgLg), M92039.1 (GgV), M92037.1 (GgB), M62903.1 (GgLr), EU124632.1 (GgM) |
| *Bos Taurus* (Bt) | NM_174566.1 (BtL), NM_001014890.1 (BtRh), NM_174567.1 (BtS), NM_001192399.1 (BtM) |
| *Homo sapiens* (Hs) | U49742.1 (HsRh), M13295.1 (HsB), M13300.1 (HsLr), K03490.1 (HsLg), BC113558.1 (HsM) |
| **Arthropoda, Hexapoda** |  |
| *Calliphora vicina* (Cv) | AJ878413.1 (Cv6), AJ878412.1 (Cv5), AJ878411.1 (Cv3), M58334.1 (Cv1) |
| *Apis mellifera* (Am) | BK005512 (AmB), BK005515 (AmL2), U26026.1 (AmL1), BK005510 (AmPter), BK005513 (AmUV) |
| *Bombyx mori* (Bm) | XM_004928431.2 (BmL1), NM_001043417.1  (BmL2), BmB, BmUV, BmPter, BmUnknown^a^ |
| *Drosophila melanogaster* (Dm) | NM_079683.2 (DmRh1), NM_079674.2 (DmRh2), NP_524411.1 (DmRh3), NP_476701.1 (DmRh4), NP_477096.1 (DmRh5), Z86118.1 (DmRh6), NP_524035.2 (DmRh7) |
| *Pediculus humanus* (Ph) | XM_002427292.1 (PhL), XP_002422743.1 (PhUV), XP_002432663.1 (PhUnknown) |
| *Acyrthosiphon pisum* (Ap) | XP_001951613.1 (ApUV1), XP_001951588.1 (ApUV2), XP_001952294.1 (ApPter), AJ489281(ApL) |
| *Bombus impatiens* (Bi) | AY655163.1 (BiUV), AY485302.1 (BiL1), AY485306.1 (BiL2) |
| *Tribolium castaneum* (Tc) | TcUV, TcL, TcPter^a^ |
| *Papilio glaucus* (Pg) | AF077189.1 (PgL1), AF077190.1 (PgL2), AF067080.1 (PgL3), AF077193.1 (PgL4), AF077191.1 (PgUV), AF077192.1 (PgB) |
| *Papilio xuthus* (Px) | AB190360.1 (PxCerebral), AB007423.1 (PxL1), AB007424.1 (PxL2), AB007425.1 (PxL3), AB028217.1 (PxB), AB028218.1 (PxUV) |
| *Danaus plexippus* (Dp) | AY605545.1 (DpL), AY605544.1 (DpB), AY605546.1 (DpUV) |
| **Arthropoda, Crustacea**  *Daphnia pulex* (Dp_) | Dp_B, Dp_UV, Dp_ND1, Dp_ND2, Dp_LA1, Dp_LA2, Dp_LA3, Dp_LA4, Dp_LA5N, Dp_LA6, Dp_LA7I, Dp_LA8, Dp_LA9, Dp_LA10, Dp_LB1, Dp_LB2, Dp_LB3, Dp_LB4, Dp_LB5, Dp_LB6, Dp_LB7, Dp_LB8, Dp_LB9, Dp_LB10, Dp_LB11, Dp_LB12, Dp_LB13, Dp_LB14, Dp_LB15, Dp_Arth1, Dp_Arth2, Dp_Arth3, Dp_Arth4, Dp_Arth5, Dp_Arth6, Dp_Arth7, Dp_Arth8, Dp_Pter1, Dp_Pter2P, Dp_Pter3, Dp_Pter4, Dp_Pter5P, Dp_Pter6, Dp_Pter7, Dp_Pter8, Dp_Pter9^b^ |
| *Odontodactylus scyllarus* (Os) | GQ221740.1 (Os1), GQ221741.1 (Os2), GQ221742.1 (Os3), GQ221743.1 (Os4), GQ221744.1 (Os5), GQ221745.1 (Os6), GQ221746.1 (Os7), GQ221747.1 (Os8), GQ221748.1 (Os9), GQ221749.1 (Os10), GQ221750.1 (Os11) |
| **Arthropoda, Chelicerata** |  |
| *Limulus polyphemus* (Lp) | L03782.1 (LpLocelli), L03781.1 (LpLeye) |
| *Hasarius adansoni* (Ha)  *Ixodes scapularis* (Is) | AB251846 (HaRh1), AB251847 (HaRh2), AB251848 (HaRh3)  IsOp1, Is2Unknown, IsOp3^c^ |
| **Mollusca, Cephalopoda**  *Todarodes pacificus* (Tp) | X70498.1 (TpRh) |
| **Annelida, Polychaeta**  *Platynereis dumerilii* (Pd) | AJ316544.1 (PdRh), AY692353.1 (PdC). |

Functional abbreviations: B, blue; C, cilliary; Lr, long-red; Lg, long-green; L, long; M, melanopsin; Pter, pteropsin; R, rhabdomeric; Rh, rhodopsin; S, short; UV, ultraviolet; V, violet. ^a^ from Velarde et al [3].^b^ from Colbourne et al [4]. ^c^ from Gulia-Nuss et al [5]

**Figure legends**

**Figure S1. Amino acid alignment of mosquito opsins and the squid rhodopsin (TpRh).** Alignment of (A) *Ae. aegypti*, (B) *An. gambiae* and (C) *Cx. quinquefasciatus* opsins and the *Todarodes pacificus* rhodopsin (TpRh). Black shading, identical residues; gray shading, similar residues. Blue residues conserved in Class A Rhodopsin-like GPCRs, orange amino acids important for opsin function, red residues potentially phosporylated and, amino acids important for G-protein interaction are boxed. The residues closest to the retinal molecule (4 Å) and the Schiff base nitrogen atom in TpRh are marked with an asterisk (*) and a dagger (†), respectively. Mosquito transmembrane (TMs) domains were predicted using TMHMM and the crystal structure of the squid rhodopsin [6]. Lines and arrows above the sequences indicate regions predicted to form α- helices and β-sheets, respectively. I 1-3, intracellular loop; E 1-3, extracellular loop.

**Figure S2. Amino acid alignment of the TM domains (TM I-VII) of *An. gambiae* and *Cx. quinquefasciatus* LW opsins.** Highlighted amino acids show positions conserved in the LW opsins. Black shading, identical residues; gray shading, similar residues, based on the similarity matrix BLOSUM62. **A**. Paralog *An. gambiae* LW opsins (*AgGPRop*). Paralogs share 59 to 100% amino acid identity in the TM domains. There are not positively selected residues on any of *A. gambiae* TM domains. **B**. Paralog *C. quinquefasciatus* LW opsins (*CqGPRop*). Paralogs share 60 to 100% an amino acid identity in the TM domains. Although shown in the Figure, *CqGPRop10* was excluded from the analysis of amino acid identity. The automatic prediction of *CqGPRop10* TM was manually curated to show TMVI and TMVII to resemble the other *C. quinquefasciatus* LW paralogs. Asterisk (*) shows the only residue (C126 on *CqGPRop5* and op6 on TMIII) identified as being under positive selection on *C. quinquefasciatus* TM domains by Fitmodel analysis.

**Figure S3. Detection of opsin transcripts in *Ae. aegypti* adult females by RT-PCR*.*** RT-PCR was performed on cDNA and gDNA (control). The expected sizes of cDNA amplicons for *AaGPRop*1-5, 7, 8-10, 12 are 729, 1,238, 599, 461, 484, 606, 274, 237, 674, 457 bp, respectively. The expected size of gDNA amplicons for *AaGPRop*1-5, 7, 8-10, 12 are 788, 1,302, 671, 461, 484, 663, 343, 12,848, 738 and 14,854 bp, respectively. Lap, Lysosomal aspartic protease housekeeping gene, 437 bp cDNA; M, molecular size marker; C. cDNA. G, gDNA.

**Figure S4. Predicted phylogenetic relationships of insect long wavelength (LW)-sensitive opsins.** The best-fit model of molecular evolution was SYM+I+G and the second was GTR, according to the Akaike Information Criterion, AIC, the corrected AICc and the Bayesian Information Criterion, BIC. The hemimetabolus insects *A. pisum* (ApL) and *P. humanus* (PhL) were employed as outgroup. Topology and branch support were estimated using maximum likelihood (A), Bayesian (B) and parsimony (C), approaches, in each case bootstrap values or posterior probabilities are shown at the node. Species abbreviations and accession numbers are shown in the legend of Figure 2 and Table S9 respectively.

**A.**

5’ **Extracellular**

*TpRh* 1 --MGRDLRDNETWWY---------------------------------------------------------------NP 15

AaGPRop1 1 --MAAFVAPHFDAW--------QSSGNMTVVDKV--------------------------------------------PP 26

AaGPRop2 1 --MAAFVEPHFDAW-------QAAGGNLTVVDKV--------------------------------------------PP 27

AaGPRop3 1 --MVALAEPHFQAWI------QSAATNVSVVDKV--------------------------------------------PA 28

AaGPRop4 1 -------MASYGAWMAAQSAGHAVASNLTVVDRV--------------------------------------------PA 29

AaGPRop5 1 -------MASYGAWMAAQSAGHAVASNLTVVDRV--------------------------------------------PA 29

AaGPRop7 1 ---MTYYGPPPNLW-------GHSVTNLTVVDKV--------------------------------------------PP 26

AaGPRop8 1 MPFEEHLSDNFTAVLRPEA--RLSAETRYLGWNV--------------------------------------------AP 34

AaGPRop9_1 1 ----MFLLNETDAAIFPMA--RTGDMPKMLGWNL--------------------------------------------PP 30

AaGPRop10 1 MKLILFFSFHFTPPIIV----RHSTATKTLIPKIDTRKLFANSQLCLSSCKRERLQSAVVLFKNPSSISLLTQFSSVFPP 76

**TMI** **I1 TMII**

TpRh 16 SIV-------------------VHPHWREFDQVPDAVYYSLGIFIGICGIIGCGGNGIVIYLFTKTKSLQTPANMFIINL 76

AaGPRop1 27 EMLHM-----------------VHPHWNQFPPMNPLWHSILGFAIFVLGVVSMLGNGCVIYIFTGTRSLRTPSNLLVVNL 89

AaGPRop2 28 EMLHM-----------------IHPHWNQFPPMNPLWHSILGFAIFVLGMVSMIGNGFVMSIFTGTPSLRTPSNLLVVNL 90

AaGPRop3 29 DMLHM-----------------VDAHWYQFPPMNPLWHSLLGFAIFVLCFISLIGNGMVIYIFTNTKTLRTPSNLLVVNL 91

AaGPRop4 30 DMLHM-----------------VDAHWYQFPPMNPLWHSLLGFAIAVLCFISVVGNGMVMYIFTNTKTLRTPSNLLVVNL 92

AaGPRop5 30 DMLHM-----------------VDAHWYQFPPMNPLWHSLLGFAIAVLCFISVVGNGMVMYIFTNTKTLRTPSNLLVVNL 92

AaGPRop7 27 EIMHL-----------------VDPHWYQFPPMNPLWHSIIGFAIFMLGMISTVGNGVVIYIFSTEKSLRTPSNLFVVNL 89

AaGPRop8 35 EDLPH-----------------IPEHWLKYPEPEASLHYLLGLLYIAFTIFALVGNGLVIWVFSSAKSLRTPSNVFVVNL 97

AaGPRop9_1 31 EQQHL-----------------VHDHWKDFPAPPYYMHLLLAMLYFVLMSVSLIGNGIVVWIFSTSKSLRNGSNMFVVNL 93

AaGPRop10 77 NSRYMALSGYSGPTIEDAFRDRINPFWLQFDPPSRTAHYILGFIYFMMMMFGLCGNLLVILMFFRFKSLRTPANYLVINL 156

**† E1 TMIII †* ** ** I2 TMIV**

TpRh 77 AFSDFTFSLVNGFPLMTISCFLKKWIFGF-AACKVYGFIGGIFGFMSIMTMAMISIDRYNVIGRPMAASKKMSHRRAFIM 155

AaGPRop1 90 AFSDFFMMFTMG-PPMVINCWHETWVFGP-FACELYAMFGSLFGCASIWTMTMIAFDRYNVIVKGLSA-KPLTNNGALLR 166

AaGPRop2 91 AFSDFLMMFTMG-PPMVINCWHETWVFGP-FACEVYACLGSLFGCASIWTMTMIAFDRYNVIVKGLAA-KPLTNNGAMLR 167

AaGPRop3 92 AFSDFLMMFTMG-PPMVYNCYHETWVLGP-FACELYGMFGSLFGCVSIWTMTMIAFDRYNVIVKGLSA-KPMGNNGALLR 168

AaGPRop4 93 AFSDFLMMFTMG-PPMVINCYYETWVFGP-FACEVYGMFGSLFGCVSIWTMTMIAFDRYNVIVNGLSG-KPLTNNGALAR 169

AaGPRop5 93 AFSDFLMMFTMG-PPMVINCYYETWVFGP-FACEVYGMFGSLFGCVSIWTMTMIAFDRYNVIVNGLSG-KPLTNNGALAR 169

AaGPRop7 90 AVSDFLMMATNA-TTMVYNCWFETWSLGL-LMCDLYAFTGSLFGCCSIWSMTMIAMDRYNVIVKGLSG-KPLTNTGAIVR 166

AaGPRop8 98 AICDFLMMVKT--PIFIYNSFTKGFTTGF-LGCQVFAFIGSLSGIGAGATNACIAYDRYNTIARPFEG--KLTHTKAIFI 172

AaGPRop9_1 94 AIFDLLMMCEM--PMFLVNSF-AGYLVGYETSCAVYAALGSLSGIGGSITNAVIAYDRYRTISNPLDG--RLNRVQSGIL 168

AaGPRop10 157 AIADFIIMLEA--PLFVYNSYHQGPATGN-VWCTIYALLGAVGGTVAIVTLTMISIDRYNVVVYPLNPKRSTTRLKVALM 233

**E2 † *** TMV ** ***

TpRh 156 IIFVWLWSVLWAIGPIF--GWGAYTLEGVLCNCSFDYISRDSTTRSNILCMFILGFFGPILIIFFCYFNIVMSVSNHEKE 233

AaGPRop1 167 ILGIWAFALFWTLAPFF--GWNRYVPEGNMTACGTDYLTTTWLNRSYIIVYAIFVYWTPLLTIIYSYTFILKAVSAHEKN 244

AaGPRop2 168 ILGIWAFALFWTLAPFF--GWNRYVPEGNMTACGTDYLTQTWLSRSYIIIYAIFVYWTPLLTIIYSYTFILKAVSAHEAQ 245

AaGPRop3 169 IFFVWGSSLAWTLAPFF--GWNRYVPEGNMSACGTDYLTDTLLSRSYILVYSIFVYFAPLLLIIYSYIFIIKAVSAHEKN 246

AaGPRop4 170 ICGVWVSTLAWTLAPFF--GWNRYVPEGNMSACGTDYLTDTFSSRSYILVYSIFVYFAPLFLIIYSYTFIIKAVSAHEKN 247

AaGPRop5 170 ICGVWVSTLAWTLAPFF--GWNRYVPEGNMSACGTDYLTDTFSSRSYILVYSIFVYFAPLFLIIYSYTFIIKAVSAHEKN 247

AaGPRop7 167 ICVCWTIGIVWGSMPML--GWNRYVPEGNMTACGTDYLTDDWFSKSYILCYSFCVYYIPLFTIIYCYVYIVKAVTVHERT 244

AaGPRop8 173 TCMVWTYTFPWAVLPLME-LWGRFVPEGYLTSCTFDYLTNTFDNRMFVGTIFTFSYVLPMSLIIYYYSQIVSHVVNHEKA 251

AaGPRop9_1 169 IFITWLWAMPFTILPVFK-IWGRYIPEGFLTTCSFDYLTDDSDTRVFVGCIFAWAYAIPMVLICYYYGRLFGHVSKHELM 247

AaGPRop10 234 IVFAWIYGLVFSVIPALDIGLSRYTPEGFLTACSFDYLERTRDARLFMFLYFIFAWVVPIIAITFCYIQILRVV------ 307

**I3 TMVI E3 TMVII**

TpRh 234 MAAMAKRLNAKELRKAQAG--ANAEMRLAKISIVIVSQFLLSWSPYAVVALLAQFGPLEWVTPYAAQLPVMFAKASAIHN 311

AaGPRop1 244 MREQAKKMNVASLRSSEAQ-QTSAEIKLAKVALVTISLWFMAWTPYLVINFTGIFKAAP-ISPLATIWGSLFAKANAVYN 270

AaGPRop2 246 MREQAKKMNVASLRSTEAN-QTSAEIKLAKVALVTISLWFMAWTPYLVINFTGIFKAAP-ISPLATIWGSLFAKANAVYN 323

AaGPRop3 247 MREQAKKMNVASLRSSEAQ-STSTEMKLAKVALVTISLWFMAWTPYLIINYTGIFKAAP-ITPLATIWGSLFAKANAVYN 324

AaGPRop4 248 MREQAKKMNVASLRSSEAQ-NTSTEMKLAKVALVTISLWFLAWTPYLIINYTGIFKASP-ISPLATIWGSLFAKANAVYN 325

AaGPRop5 248 MREQAKKMNVASLRSSEAQ-NTSTEMKLAKVALVTISLWFLAWTPYLIINYTGIFKASP-ISPLATIWGSLFAKANAVYN 325

AaGPRop7 245 MREQAKRMNIQSLRQGDDG--KAAEMKLAKIALVTISLWFLAWTPYTIINYTGVFKMAS-LTPLATIWGSVFAKCSSVYN 321

AaGPRop8 252 LREQAKKMNVDSLRSNQNQANTSVEVRIAKAAITVCFLFVASWTPYAVLALIGAFGDKTLLTPGVTMFPACACKFVACLD 331

AaGPRop9_1 248 LKNQARKMNVESLASNRNEKAQSVEIRIARAAFTIFFLFVCAWTPYAIVAMIGAYGDRTLLTPFFTMIPAVCCKIVSCLD 327

AaGPRop10 307 -------IGANSIQSSKNK--SKTEVKLAGVVIGIIGLWFIAWTPYAIVAMMGVFGYESLLSPLGSMVPAILAKTAACID 378

**3’ Intracellular**

TpRh 312 PMIYSVSHPKFREAISQTFPWVLTC-----------------CQFDDKETEDDKDAETEIPA----GESSDAAPSADAAQ 370

AaGPRop1 271 PIVYGISHPKYRAALTQKFPA-LSC--------------------TDAPAASNSDDNQSTVS----GATTATDMREQAKK 325

AaGPRop2 324 PIVYGISHPKYRAALYQRHPW-LSC----------------------QDAQESSHDNQSTVS----GATTATEEKA---- 372

AaGPRop3 325 PIVYGISHPKYRAALYQKFPS-LSC--------------------TDAAD-----DSQSMAS----GTTTVVQEEKPSA- 373

AaGPRop4 326 PIVYGISHPKYRAALYQKFPS-LSC--------------------TDPAD-----DTQSVAS----GTTTVVSEKSEKT- 374

AaGPRop5 326 PIVYGISHPKYRAALYQKFPS-LSC--------------------TDPAD-----DSQSVAS----GTTTVVSEKSEKT- 374

AaGPRop7 322 PIVYGISHPKYRAALVRRFPA-LGC--------------------GDASGGADAKSMASEVSAVSGGGSTGAGMETTAA- 379

AaGPRop8 332 PYVYAISHPRYRVELQKRLPW-LAI--------------------TESLPS----DSASNVT----DATTANTSAPASS- 381

AaGPRop9_1 328 PWVYAISHPKYRQELERRLPW-MGI--------------------REPADNVSTTDSKHTVV----SESLPVGPNGID-- 380

AaGPRop10 379 PYFYAMNHPRYRQELRKMFG--LNQQDLGNSQYQTSRYTRNASRMDDSEGGASERVTIGRQP----GKTTTDEPEPSQQT 452

TpRh 371 MKEMMAMMQKMQQQQAAYPPQGYAPPPQGYPPQGYPPQGYPPQGYPPQGYPPPPQGAPPQGAPPAAPPQGVDNQAYQA 448

AaGPRop1 326 MNVASLRSSEAQQTSAEIKLAKVALVTISLWFMAWTPYLVINFTGEKA------------------------------ 373

AaGPRop2 372 ------------------------------------------------------------------------------ 372

AaGPRop3 373 ------------------------------------------------------------------------------ 373

AaGPRop4 374 ---------------------------------------------ESA------------------------------ 377

AaGPRop5 374 ---------------------------------------------ESA------------------------------ 377

AaGPRop7 379 ------------------------------------------------------------------------------ 379

AaGPRop8 381 ------------------------------------------------------------------------------ 381

AaGPRop9_1 380 ------------------------------------------------------------------------------ 380

AaGPRop10 453 EQGPQPTYSKNLAANSRGALQRAQSSISAADDTSLSVSIDLTETNPNSNH---------------------------- 502

**B.**

5’ **Extracellular** TMDI TpRh 1 -------------------MGRDLR--DNETW------------W----------YNPSIV--VHPHWREFDQVPDAVYY 35

AgGPRop1 1 ------------------MAAFVEP--HFDAWTQG-SGNMSVVDK----------VPPEMLHMVHPHWNQFPPMNPLWHS 49

AgGPRop3 1 ------------------MAAFVEP--HFDAWTQS-GGNMSVVDK----------VPPEMLHMVHPHWNQFPPMNPLWHS 49

AgGPRop4 1 ------------------MAAFVEP--HFDAWTQS-GGNMSVVDK----------VPPEMLHMVHPHWNQFPPMNPLWHS 49

AgGPRop5 1 ----------MMDHRPVGIFGPKSP--QALTWTIS-VANLTVVDK----------VPPEMLHLVDTYWYQFPPLETKWHA 57

AgGPRop6 1 ------------------MAAFAEP--HFSAWTQTVVSNVTVVDK----------VPPEMLHMVDAHWYQFPPMNPLWHS 50

AgGPRop7 1 -------------------MPYYGPMQQPGLWGQP-VANLTVVDK----------VPPEIMHLVDPHWSQFPPMNPLWHS 50

AgGPRop8 1 --MGLVQLDNQTAYRPEALIGADQSGLRYLGW------------N----------VPPEELVHIPEHWLQFPEPEASLHY 56

AgGPRop9? 1 MFLGNESISEGAMLMPMARTAGEMP--KLLGW------------N----------LPPEEQYLVHDHWKGFPSPPYYMHL 56

AgGPRop10 1 -----MGRQGSGNAVRISPSSRNQP--YFSSAHLSFVVPFPVHSKYVVRSGYVLPVDPLFVAKINPFWLRFDPPSAGEHY 73

**I1 TMII † E1 TMIII †* ***

TpRh 36 SLGIFIGICGIIGCGGNGIVIYLFTKTKSLQTPANMFIINLAFSDFTFSLVNGFPLMTISCFLKKWIFGFAACKVYGFIG 115

AgGPRop1 50 ILGFAIFMLGMISMTGNGCVMYIFTNTKSLRTPSNLLVVNLAFSDF-FMMFTMGPPMVINCWHETWTFGPFACELYAMLG 128

AgGPRop3 50 ILGFAIFMLGMISMTGNGCVMYIFTNTKSLRTPSNLLVVNLAFSDF-FMMFTMGPPMVINCWHETWTFGPFACELYAMLG 128

AgGPRop4 50 ILGFAIFMLGMISMTGNGCVMYIFTNTKSLRTPSNLLVVNLAFSDF-FMMFTMGPPMVINCWHETWTFGPFACELYAMLG 128

AgGPRop5 58 ALATTIGLLALISIVGNGCVILIFSSTKGLRTPSNLMVINLAFADF-MMMFTMAPPLIINSYHETWVFGPLMCEIYGMFG 136

AgGPRop6 51 ILGFAIFVLGVVSIIGNGCVIYIFTNTKALRTPSNLLVVNLAFSDF-LMMFTMAPPMVINCWHETWVFGPFACELYAMLG 129

AgGPRop7 51 IIGFVIFVLGVVSIIGNGMVIYIFSTAKSLRTPSNLFIVNLALSDF-LMMGTNAFTMVYNCWFETWSLGLLMCDLYAFFG 129

AgGPRop8 57 LLGLLYIAFTIFSLVGNGLVIWIFIAAKSLRTPSNVFVINLAICDF-FMMAKT-PIFIYNSFTKGFTLGNLGCQIFGFVG 134

AgGPRop9? 57 MLAMIYFVLMNTSLIGNGIVLWIFGTSKSLRNGSNMFIINLAIFDL-LMMCEM-PMFLVNSFSERLVGYGVGCSVYAALG 134

AgGPRop10 74 GLAVFYFLMMLFGVIGNALVVFMFYRYRSLRTPANYLVINLAVADF-IIMMEA-PMFIYNSIHQGPALGSIGCTVYALMG 151

*** ** I2 TMIV E2 † * ***

TpRh 116 GIFGFMSIMTMAMISIDRYNVIGRPMAASKKMSHRRAFIMIIFVWLWSVLWAIGPIF--G-WGAYTLEGVLCNCSFDYIS 192

AgGPRop1 129 SLFGCASIWTMTMIAFDRYNVIVKGLAG-KPMTNNGALLRILGVWVFALFWTLAPLF--G-WNRYVPEGNMTACGTDYLT 204

AgGPRop3 129 SLFGCASIWTMTMIAFDRYNVIVKGLAG-KPMTNNGALLRILGVWVFALFWTLAPLF--G-WNRYVPEGNMTACGTDYLT 204

AgGPRop4 129 SLFGCASIWTMTMIAFDRYNVIVKGLAG-KPMTNNGALLRILGVWVFALFWTLAPLF--G-WNRYVPEGNMTACGTDYLT 204

AgGPRop5 137 SLSGCVSIWSMTMIAFDRYTVIVKGLSA-KPLTYVGSVMRILFVWANSLVWTLAPLF--G-WNRYVPEGNMSACGTDYLS 212

AgGPRop6 130 SLFGCASIWTMTMIAFDRYNVIVKGLAG-KPMTNNGALLRILGIWAFSLLWTLAPLF--G-WNRYVPEGNMTACGTDYLS 205

AgGPRop7 130 SLFGCCSIWTMTMIALDRHNVIVHGLSG-KPLTNTGAILRILLCWLIGVVWGILPML--G-WNRYVPEGNMTACGTDYLT 205

AgGPRop8 135 SLTGIGAGATNALIAYDRYNTITRPFEG-R-LTQTKAIIFICLIWAYTIPWGVLPLL--EIWGRYVPEGFLTSCTFDYLS 210

AgGPRop9? 135 SMSGIGGAISNAVIAFDRYRTISNPLDG-R-LSRVQAGLLICLTWLWTMPFTLLPLF--EIWGRYIPEGYLTTCSFDYLT 210

AgGPRop10 152 AVGGTVAIATLTVISIDRYNVVVYPLNPNRSTTKLKCYFLIAFTWAYGLLFASFPALEIG-LSRYTAEGYLTACSFDYLD 230

**TMV ** * I3 TMVI**

TpRh 193 RDSTTRSNILCMFILGFFGPILIIFFCYFNIVMSVSNHEKEMAAMAKRLNAKELRKAQAGA--NAEMRLAKISIVIVSQF 270

AgGPRop1 205 QTWLSRSYIIIYAIFVYWLPLLTIIYSYTFILKAVSAHEKNMREQAKKMNVASLRTQEAQN-TSTEMKLAKVALVTISLW 283

AgGPRop3 205 QTWLSRSYIIIYAIFVYWLPLLTIIYSYTFILKAVSAHEKNMREQAKKMNVASLRTQEAQN-TSTEMKLAKVALVTISLW 283

AgGPRop4 205 QTWLSRSYIIIYAIFVYWLPLLTIIYSYTFILKAVSAHEKNMREQAKKMNVASLRTQEAQN-TSTEMKLAKVALVTISLW 283

AgGPRop5 213 KDWISVSYIYAYSVFVYWLPLLLIIYCYTYILKAVSAHERNMREQAKKMNVASLRSSDASK-TNAEIKLAKVALVTITLW 291

AgGPRop6 206 QDFTSRSYILIYSGFVYYLPLFSIIYSYIYIIQAVSAHEKNMREQAKKMNVASLRSQEAQN-TSTEMKLAKVALVTISLW 284

AgGPRop7 206 DDWFHKSYILVYSVFVYYTPLFTIIYAYFFIIKAVSAHEKNMREQAKRMNVQSLRSSDDGK--STEMKLAKVALVTISLW 283

AgGPRop8 211 GTFDTRLFVASIFTFSYVLPMSLIIYYYSQIVSHVVNHEKSLREQAKKMNVESLRSNQNQKDASVEIRIAKAAITVCFLF 290

AgGPRop9? 211 DDPDTRVFVGCIFTWAYVIPMIFICYFYARLFGHVRQHEMMLKNQARKMNVESLTANRSEKAQAVEMRIAKAAFTIFFLF 290

AgGPRop10 231 RTYKARVFMFVYFVFAWLIPFAIISYCYARILIAV-------------INANAIQSSKSKN--KTEVKLAGVVVGIIGLW 295

*** * E3 TMVII**

TpRh 271 LLSWSPYAVVALLAQFGPLEWVTPYAAQLPVMFAKASAIHNPMIYSVSHPKFREAISQTFPWVLTCCQFDDKETEDDKDA 350

AgGPRop1 284 FMAWTPYLVINFTGIFKAAP-ISPLATIWGSLFAKANAVYNPIVYGISHPKYRAALYQKFPS-LSC---QDA-PVDDGQS 357

AgGPRop3 284 FMAWTPYLVINFTGIFKAAP-ISPLATIWGSLFAKANAVYNPIVYGISHPKYRAALYQKFPS-LSC---QDA-PVDDGQS 357

AgGPRop4 284 FMAWTPYLVINFTGIFKAAP-ISPLATIWGSLFAKANAVYNPIVYGISHPKYRAALYQKFPS-LSC---QDA-PVDDGQS 357

AgGPRop5 292 FMAWTPYLVINYAGIIDSSP-ISPLQTIWGSVFAKANAVYNPIVYGISHPKYRAALYKRFPA-LQL---RKE-NATDQNS 365

AgGPRop6 285 FMAWTPYLVINYTGIFKAAP-ISPLATIWGSLFAKANAVYNPIVYGISHPKYRAALYQKFPS-LSC---QDN--SDDGQS 357

AgGPRop7 284 FMAWTPYTVINYTGVFKTAS-ITPLATIWGSVFAKANAVYNPIVYGISHPKYRAALLRRFPS-LAC---SDG-PPADDKS 357

AgGPRop8 291 VASWTPYAVLALIGAFGDKSLLTPGVTMFPACACKFVACLDPYVYAISHPRYRIELQKRLPW-LAI---TETLPAENAST 366

AgGPRop9? 291 VCAWTPYAIVTMIGAFGDRTMLTPFVTMVPAVCCKIVSCLDPWVYAISHPKYRQELERRLPW-MGI---KEA---DDSVS 363

AgGPRop10 296 FAAWTPYAVVAMMGVFGYEQYLTPLNSMIPAVFAKIAASIDPYFYAMNHPRYRQMLERMFCN-RGA---DQGNSQYQTSH 371

**3’ Intracellular**

TpRh 351 ETEIPAGESSDAAPSADAAQMKEMMAMMQKMQQQQAAYPPQGYAPPPQGYPPQGYPPQGYPPQGYPPQGYPPPPQGAPPQ 430

AgGPRop1 358 VASGATQASDEKA------------------------------------------------------------------- 370

AgGPRop3 358 VASGATQASDEKA------------------------------------------------------------------- 370

AgGPRop4 358 VASGATQASDEKA------------------------------------------------------------------- 370

AgGPRop5 366 VASCTTAADQCET------------------------------------------------------------------- 378

AgGPRop6 358 VASAATGVSEEKPAA----------------------------------------------------------------- 372

AgGPRop7 358 LASEASGITSAGNPTTA--------------------------------------------------------------- 374

AgGPRop8 367 CTEQQDGNATTQS------------------------------------------------------------------- 379

AgGPRop9? 364 TTES---------------------------------------------------------------------------- 367

AgGPRop10 372 YTRGASRGGDSEGGGGEESGGGGGVGRAPGGGNAGLGRGGTVRGGGGGGRLIAGKGGGGANATGSTGGGGVKALKKQISN 451

TpRh 431 GAPPAAPPQGVDNQAYQA 448

AgGPRop1 370 ------------------ 370

AgGPRop3 370 ------------------ 370

AgGPRop4 370 ------------------ 370

AgGPRop5 378 ------------------ 378

AgGPRop6 372 ------------------ 372

AgGPRop7 374 ------------------ 374

AgGPRop8 379 ------------------ 379

AgGPRop9? 367 ------------------ 367

AgGPRop10 452 GDETSLEVSLEM------ 463

**C.**

5’ **Extracellular** TMDI

TpRh 1 --MGRDLRDNETWWYNPSI--------------------VVHPHWREFDQVPDAVYYSLGIFIGICG-----------II 47

CqGPRop1 1 ---MSYYGPPPSIWGHPIS------NMTVVDKVPPEILHLVDPHWYQFPPMNPLWHSIIGFAIFVLG-----------II 60

CqGPRop2 1 ----MFLLNETDAVLLPAARTGGEMVKLLGWNLPPEQMHLVHEHWKDFPAPPYFMHLLLALIYFVLM-----------NV 65

CqGPRop3 1 MPFLEHLSDNYTAVLRPEARLSAE-TRYLGWNVAPEDLPHIPEHWLKYPEPEASLHYLLGLLYIAFT-----------IF 68

CqGPRop4 1 --MPQDCTQTTKGILHPA-------SQEIPWK------------WKGKDQVLEVLLQIFSWTTSVVCGCRMWMASTASTA 59

CqGPRop5 1 --MAAFAEPHFDAWQAGAG------NMSVVDMVAPEMLHMVHPHWNQFPPMNPLWHSILGFAIFCLG-----------MV 61

CqGPRop6 1 --MAAFVEPHFDAYQAGNG------NMTVVDMVSPDMLHMVHPHWNQFPPMNPLWHSILGFAIFCLG-----------MV 61

CqGPRop7 1 -MLGEPSLPMALSW-----GSGPVSNMTVVDKVLPDMLHLVDAHWYQFPPMNPLWHSILGFAIFVLG-----------CV 63

CqGPRop8 1 --MASY-----AAWNAAQSGLGAMVNLTVVDRVPADMLHMVDAHWYQFPPMNPLWHSILGFAIFVLC-----------FI 62

CqGPRop9 1 --MASY-----AAWNAAQSGLGAMVNLTVVDRVPADMLHMVDAHWYQFPPMNPLWHSILGFAIFVLC-----------FI 62

CqGPRop10? 1 --MASY----AAAWKAAEAVA----NLTVVDKVPADMLHMVDAHWYQFPPMNPLWHAMLGWAIFFLC-----------LI 59

CqGPRop11 1 ----MFLVNETDAVLLPAARTGEM-VKLLGWNLPPEQMHLVHEHWKDFPAPPYFMHLLLALIYFVLM-----------NL 64

CqGPRop13 1 --MASY-----AAWTAVKAGVGAAMNLTVVDKVPADMLHMVDAHWYQFPPMNPLWHAMLGWAIFFLC-----------LI 62

**I1 TMII**

TpRh 48 GCGGNGIVIY---------------------------------------LFTKTKSLQTPANMFIINLAFSDFTFSLVNG 88

CqGPRop1 61 SVIGNGMVIY---------------------------------------IFSTAKGLRTPSNLFVVNLAMSDFLMMMTNA 101

CqGPRop2 66 SLIGNGIVVW---------------------------------------IFTTSKSLRNGSNMFIVNLAIFDLLMMCEM- 105

CqGPRop3 69 ALVGNGLVIW---------------------------------------IFSSAKSLRTPSNVFIVNLAICDFLMMVKT- 108

CqGPRop4 60 DLTGGGYVAYGRDLHRSWELKGKDHQLCWSEPGFEPRSTAYETEALPLGYVARFKSLRTPANYLVINLAVADFLIMLEA- 138

CqGPRop5 62 SMIGNGCVIS---------------------------------------IFTGTKSLKTPSNLLVVNLAFSDFLMMFTMG 102

CqGPRop6 62 SMIGNGCVIS---------------------------------------IFTSTKSLKTPSNLLVVNLAFSDFLMMFTMG 102

CqGPRop7 64 SVIGNGCVVS---------------------------------------IFTGTKSLRTPSNMLVINLAFSDFLMMFTMA 104

CqGPRop8 63 SLVGNGCVVY---------------------------------------IFTNTKTLRTPSNLLVVNLAFSDFLMMFTMG 103

CqGPRop9 63 SLVGNGCVVY---------------------------------------IFTNTKTLRTPSNLLVVNLAFSDFLMMFTMG 103

CqGPRop10? 60 SVIGNGMVVY---------------------------------------IFTNTRTLRTPSNLLVVNLAFSDFLMMFTMG 100

CqGPRop11 65 SLIGNGIVVW---------------------------------------IFTTSKSLRNGSNMFIVNLAIFDLLMMCEM- 104

CqGPRop13 63 SLIGNGMVIN---------------------------------------IFTSTKTLKTPSNLLVVNLAFSDFLMMFTMG 103

**E1 TMIII I2 TMIV**

†* ** **

TpRh 89 FPLMTISCFLKKWIFGFAACKVYGFIGGIFGFMSIMTMAMISIDRYNVIGRPMAASKKMSHRRAFIMIIFVWLWSVLWAI 168

CqGPRop1 101 -FTMVYNCWYETWQLGVLMCDLYAFTGSLFGCCSIWTMTMIALDRYNVIVKGLAG-KPLTNSGAVLRILVCWTIGVVWGI 179

CqGPRop2 105 -PMFLVNAFSERLVGYETGCAIYAALGSVSGIGGAISNAVIAYDRYRTISNPLEG--RMNRTKASLFVVMTWLWTVPFTV 182

CqGPRop3 108 -PIFIYNSFTRGFTTGYLGCQIFAFIGSLSGIGAGATNACIAYDRYNTIARPFEG--KLTHTKAIFITCLVWAYTIPWGL 185

CqGPRop4 138 -PIFVYNSYHLGPAFGNT-CTIYSLLGAIGGTVAIMTLTMISVDRYNVVVYPLNPNRSTTRLKVMLMIVFTWIYALVFSL 216

CqGPRop5 102 -PPMVINCWHQTWVFGPFACELYACLGSLFGCASIWTMTLIAFDRYNVIVKGLAA-KPMTNSGAMVKILMVWAFALFWTL 180

CqGPRop6 102 -PPMVINCWHQTWSFGPFACELYACLGSLFGCASIWTMTLIAFDRYNVIVKGLAA-KPMTNSGAMVKILLVWAFALFWTL 180

CqGPRop7 104 -PPMVMSCYHETWVFGPLMCQVYAMLGSLFGCVSIWSMTMIAFDRYNVIVKGLSA-TPMTFNNALLKIAFIWANALIWTL 182

CqGPRop8 103 -PPMVINCYHETWVFGPFACELYGMLGSLFGCVSIWSMTLIAFDRYNVIVKGLSA-KPMTNNGALLRIFLVWASCLAWTI 181

CqGPRop9 103 -PPMVINCYHETWVFGPFACELYGMLGSLFGCVSIWSMTLIAFDRYNVIVKGLSA-KPMTNNGALLRIFLVWASCLAWTI 181

CqGPRop10? 100 -PPMVINCYYETWVFGAFACEVYGMFGSLFGCVSIWTMTMIAFDRYNVIVKGLSA-KPLTNNGAMVQIFGVWIASLGWTL 178

CqGPRop11 104 -PMFLVNAFSERLVGYETGCAIYAALGSVSGIGGAISNAVIAYDRYRTISNPLEG--RMNRVQASLFVLLTWLWTLPFTV 181

CqGPRop13 103 -PPMVMNCYYETWVFGPFACEVYAMCGSLFGCISIWTMTMIAFDRYNVIVNGISA-EPLTNKGATIRIFAIWATSFAWTL 181

**E2†*** TMV ** * I3**

TpRh 169 GPIFG--WGAYTLEGVLCNCSFDYISRDSTTRSNILCMFILGFFGPILIIFFCYFNIVMSVSNHEKEMAAMAKRLNAKEL 246

CqGPRop1 180 LPMLG--WNRYVPEGNMTACGTDYLTEDWFHKSYILAYSFFVYYVPLFTIIYSYVYIVKAVSEHEKSMRDQAKRMNMQSL 257

CqGPRop2 183 MPMFNI-WGRYIPEGYLTTCSFDYLTDDSDTRVFVGCIFAWAYAIPMVFICYFYTRLFGHVRQHENMLKNQARKMNIESL 261

CqGPRop3 186 LPLMEI-WGRFVPEGYLTSCTFDYLTNTFDNRMFVGTIFTFSYVLPMSLIIYYYSQIVSHVVNHEKALREQAKKMNVDSL 264

CqGPRop4 217 MPALEIGLSRYTPEGFLTACSFDYLDRGWDARVFMFMYFVFAWVIPFLTISYCYVAILRVV-------------VGAGSI 283

CqGPRop5 181 APFFG--WNRYVPEGNMTACGTDYLTQTWLSRSYIIVYAIFVYWLPLLTIIYSYTFILKAVSAHEEQMREQAKKMNVASL 258

CqGPRop6 181 APFFG--WNRYVPEGNMTACGTDYLTQTWLSRSYIIVYAVFVYWLPLLTIIYSYTFILKAVSAHEEQMREQAKKMNVASL 258

CqGPRop7 183 APMFG--WNRYVPEGNMTACGTDYLTQDIVSTSYIMVYSLFVYWLPLLMIIYSYTFILKAVADHEKNMREQAKKMNVASL 260

CqGPRop8 182 PPFFG--WNRYVPEGNMSACGTDYLTDTFLSRSYILVYSVFVYFAPLLMIIYSYIFIIKAVSAHEKNMREQAKKMNVASL 259

CqGPRop9 182 PPFFG--WNRYVPEGNMSACGTDYLTDTFLSRSYILVYSVFVYFAPLLMIIYSYIFIIKAVSAHEKNMREQAKKMNVASL 259

CqGPRop10? 179 APFLG--WNRYVPEGNMSACGTDYLTDTMLSRSYILVYSMFVYFAPLLLIIYSYIFIIKAVSAHEKNMREQAKKMNVASL 256

CqGPRop11 182 LPMFNI-WGRYIPEGYLTTCSFDYLTDDSDTRVFVGCIFTWAYAIPMVFICYFYTRLFGHVRQHENMLKNQARKMNIESL 260

CqGPRop13 182 APFFG--WNRYVPEGNMSACGTDYLTDTFSSRSYILMYSVFVYFAPLFLIIYSYIFIIKAVAAHEKNMREQAKKMNVASL 259

**TMVI * * E3 TMVII**

TpRh 247 RKA--QAGANAEMRLAKISIVIVSQFLLSWSPYAVVALLAQFGPLEWVTPYAAQLPVMFAKASAIHNPMIYSVSHPKFRE 324

CqGPRop1 258 RQG--DDGKAAEMKLAKIALVTISLWFMAWTPYTIINYTGVFKTAN-ISPLATIWGSVFAKANAVYNPIVYGISHPKYRA 334

CqGPRop2 262 AANRNANAEAAEIRIAKAAFTIFFLFVCAWTPYAFVAMIGAFGDKTILTPFFTMIPAMCCKIVSCLDPWVYAISHPRYRQ 341

CqGPRop3 265 RSNQNQANSSVEVRIAKAAITVCFLFVASWTPYAVLALIGAFGDKSLLTPGVTMFPACACKFVACLDPYVYAISHPRYRV 344

CqGPRop4 284 QSS--KNKNKQEVKLAGVVIGIIGLWFIAWTPYAVVAMLGVFGYEHLLTPLGSMIPAILAKTASCIDPYFYAMNHPRFRQ 361

CqGPRop5 259 RSS-EAQQTSAEIKLAKVALMTISLWFMAWTPYLVINFTGIFKAAP-ISPLATIWGSLFAKANAVYNPIVYGISHPKYRA 336

CqGPRop6 259 RSS-EAQQTSAEIKLAKVALVTISLWFMAWTPYLVINFTGVFKAAP-ITPLATIWGSLFAKANAVYNPIVYGISHPKYRA 336

CqGPRop7 261 RSS-DAAKQSAEIRLAKVALVTISLWFLAWTPYLVINYTGIFKAAP-ISPLATIWGSLFAKANAVYNPIVYGISHPKYRA 338

CqGPRop8 260 RSS-EAQNTSTEMKLAKVALVTISLWFMAWTPYLIINYTGIFKAAP-ITPLATIWGSLFAKANAVYNPIVYGISHPKYRA 337

CqGPRop9 260 RSS-EAQNTSTEMKLAKVALVTISLWFMAWTPYLIINYTGIFKAAP-ITPLATIWGSLFAKANAVYNPIVYGISHPKYRA 337

CqGPRop10? 257 RSQ-ETQSTSTEMKL-----------------YLIINYTGIFKAAP-ITPLATIWGSLFAKANAVYNPIVYGISHPKYRA 317

CqGPRop11 261 AANRNVNAEAAEIRIAKAAFTIFFLFVCAWTPYAIVTMIGAFGDKTMLTPFFTMVPAMCSKVVSCLDPWVYAISHPKYRQ 340

CqGPRop13 260 RSS-EAQNTSTEMKLAKVALVTISLWFMAWTPYLIINYTGIFKAAP-ITPLATIWGSLFAKANAVYNPIVYGISHPKYRA 337

**3’ extracellular**

TpRh 325 AISQTFPWVLTCCQFDDKETEDDKDAETEIPAGESSDAAPSADAAQMKEMMAMMQKMQQQQAAYPPQGYAPPPQGYPPQG 404

CqGPRop1 335 ALYKRFPS-LSCQDV---VAADDKSLASEASAVTTSNSGDTASA------------------------------------ 374

CqGPRop2 342 ELEKRLPW-LGIKE----APDNVSTTESKQTVVAEPAANAET-------------------------------------- 378

CqGPRop3 345 ELQKRLPW-LAITET---LPSDTASTTTEATTTNATASS----------------------------------------- 379

CqGPRop4 362 ELRKMFG-----KEQEMNHSQYQTSRYTRNASRNDSEAGPSERVQLGRAPGKDADPIPAVSSSVAQPNYSQNLASNRKGG 436

CqGPRop5 337 ALYQRYPG-LSC------QSDNSSNDSQSVATTAEEEKA----------------------------------------- 368

CqGPRop6 337 ALYQRYPG-LSCQSDN--SSNDTQSVASGATTASDEKA------------------------------------------ 371

CqGPRop7 339 ALYKKYPS-LACSTD---SVSDDQSVASGATVASDNNNA----------------------------------------- 373

CqGPRop8 338 ALYQKFPS-LSCTD----AVDDSQSVTSGTTTVVNEEKPSA--------------------------------------- 373

CqGPRop9 338 ALYQKFPS-LSCTD----AVDDSQSVTSGTTTVVNEEKPSA--------------------------------------- 373

CqGPRop10? 318 ALYQKFPS-LSCSD----SVEDTQSMASAETTVAEKTAA----------------------------------------- 351

CqGPRop11 341 ELERRLPW-LGIKE----APDNVSTTESKQTVISDPPAAAEN-------------------------------------- 377

CqGPRop13 338 ALYKKFPA-LSCTD----SADDSQSVASGETVITEKAEKIEA-------------------------------------- 374

TpRh 405 YPPQGYPPQGYPPQGYPPPPQGAPPQGAPPAAPPQGVDNQAYQA 448

CqGPRop1 374 -------------------------------------------- 374

CqGPRop2 378 -------------------------------------------- 378

CqGPRop3 379 -------------------------------------------- 379

CqGPRop4 437 LQRAQSSISAADDTSLSCSIDLTETQPNNH-------------- 466

CqGPRop5 368 -------------------------------------------- 368

CqGPRop6 371 -------------------------------------------- 371

CqGPRop7 373 -------------------------------------------- 373

CqGPRop8 373 -------------------------------------------- 373

CqGPRop9 373 -------------------------------------------- 373

CqGPRop10? 351 -------------------------------------------- 351

CqGPRop11 377 -------------------------------------------- 377

CqGPRop13 374 -------------------------------------------- 374

Figure S1.

**A.**

**TMI TMII TMIII**

*AgGPRop1* ILGFAIFMLGMISMTGNGCVMYI—-LLVVNLAFSDFFMMFTMGPPMVI-ELYAMLGSLFGCA

*AgGPRop3* ILGFAIFMLGMISMTGNGCVMYI—-LLVVNLAFSDFFMMFTMGPPMVI-ELYAMLGSLFGCA

*AgGPRop4* ILGFAIFMLGMISMTGNGCVMYI—-LLVVNLAFSDFFMMFTMGPPMVI-ELYAMLGSLFGCA

*AgGPRop5* -LATTIGLLALISIVGNGCVILIF-LMVINLAFADFMMMFTMAPPLII-EIYGMFGSLSGCV

*AgGPRop6* ILGFAIFVLGVVSIIGNGCVIYI—-LLVVNLAFSDFLMMFTMAPPMVI-ELYAMLGSLFGCA

*AgGPRop7* IIGFVIFVLGVVSIIGNGMVIYI—-LFIVNLALSDFLMMGTNAFTMVY-DLYAFFGSLFGCC

**TMIV TMV TMVI**

*AgGPRop1* SIWTMTMIAF-ALLRILGVWVFALFWTLAPLFGW-YIIIYAIFVYWLPLLTIIYSYTF-VALV

*AgGPRop3* SIWTMTMIAF-ALLRILGVWVFALFWTLAPLFGW-YIIIYAIFVYWLPLLTIIYSYTF-VALV

*AgGPRop4* SIWTMTMIAF-ALLRILGVWVFALFWTLAPLFGW-YIIIYAIFVYWLPLLTIIYSYTF-VALV

*AgGPRop5* SIWSMTMIAF-SVMRILFVWANSLVWTLAPLFGW-Y—-AYSVFVYWLPLLLIIYCYTY-ILLV

*AgGPRop6* SIWTMTMIAF-ALLRILGIWAFSLLWTLAPLFGW-YILIYSGFVYYLPLFSIIYSYIY-VALV

*AgGPRop7* SIWTMTMIAL-AILRILLCWLIGVVWGILPMLGW-YILVYSVFVYYTPLFTIIYAYFF-VALV

**TMVII**

*AgGPRop1* TISLWFMAWTPYLVINFTG-LATIWGSLFAKANAVYNPIVYGI--

*AgGPRop3* TISLWFMAWTPYLVINFTG-LATIWGSLFAKANAVYNPIVYGI--

*AgGPRop4* TISLWFMAWTPYLVINFTG-LATIWGSLFAKANAVYNPIVYGI--

*AgGPRop5* TITLWFMAWTPYLVINYAGII-TIWGSVFAKANAVYNPIVYGISH

*AgGPRop6* TISLWFMAWTPYLVINYTG-LATIWGSLFAKANAVYNPIVYGI--

*AgGPRop7* TISLWFMAWTPYTVINYTG-LATIWGSVFAKANAVYNPIVYGI--

**B.**

**TMI TMII TMIII**

*CqGPRop1* IIGFAIFVLGIISVIGNGMVIYI-LFVVNLAMSDFLMMMTNAFTMVY-DLYAFTGSLFGC

*CqGPRop7* ILGFAIFVLGCVSVIGNGCVVSI-MLVINLAFSDFLMMFTMAPPMVM-QVYAMLGSLFGC

*CqGPRop5* ILGFAIFCLGMVSMIGNGCVISI-LLVVNLAFSDFLMMFTMGPPMVI-ELYACLGSLFGC

*CqGPRop6* ILGFAIFCLGMVSMIGNGCVISI-LLVVNLAFSDFLMMFTMGPPMVI-ELYACLGSLFGC

*CqGPRop9* ILGFAIFVLCFISLVGNGCVVYI-LLVVNLAFSDFLMMFTMGPPMVI-ELYGMLGSLFGC

*CqGPRop8* ILGFAIFVLCFISLVGNGCVVYI-LLVVNLAFSDFLMMFTMGPPMVI-ELYGMLGSLFGC

*CqGPRop13* LGWAIFFLCLISLIGNGMVINIF-LLVVNLAFSDFLMMFTMGPPMVM-EVYAMCGSLFGC

*CqGPRop10* LGWAIFFLCLISVIGNGMVVYIF-LLVVNLAFSDFLMMFTMGPPMVI-EVYGMFGSLFGC

*

**TMIV TMV**

*CqGPRop1* CSIWTMTMIAL-AVLRILVCWTIGVVWGILPMLGW-YILAYSFFVYYVPLFTIIYSYVY-

*CqGPRop7* VSIWSMTMIAF-ALLKIAFIWANALIWTLAPMFGW-YIMVYSLFVYWLPLLMIIYSYTF-

*CqGPRop5* ASIWTMTLIAF-AMVKILMVWAFALFWTLAPFFGW-YIIVYAIFVYWLPLLTIIYSYTF-

*CqGPRop6* ASIWTMTLIAF-AMVKILLVWAFALFWTLAPFFGW-YIIVYAVFVYWLPLLTIIYSYTF-

*CqGPRop9* VSIWSMTLIAF-ALLRIFLVWASCLAWTIPPFFGW-YILVYSVFVYFAPLLMIIYSYIF-

*CqGPRop8* VSIWSMTLIAF-ALLRIFLVWASCLAWTIPPFFGW-YILVYSVFVYFAPLLMIIYSYIF-

*CqGPRop13* ISIWTMTMIAF-ATIRIFAIWATSFAWTLAPFFGW-YILMYSVFVYFAPLFLIIYSYIF-

*CqGPRop10* VSIWTMTMIAF-AMVQIFGVWIASLGWTLAPFLGW-YILVYSMFVYFAPLLLIIYSYIF-

**TMVI TMVII**

*CqGPRop1* IALVTISLWFMAWTPYTIINYTG-LATIWGSVFAKANAVYNPIVYGI

*CqGPRop7* VALVTISLWFLAWTPYLVINYTG-LATIWGSLFAKANAVYNPIVYGI

*CqGPRop5* VALMTISLWFMAWTPYLVINFTG-LATIWGSLFAKANAVYNPIVYGI

*CqGPRop6* VALVTISLWFMAWTPYLVINFTG-LATIWGSLFAKANAVYNPIVYGI

*CqGPRop9* VALVTISLWFMAWTPYLIINYTG-LATIWGSLFAKANAVYNPIVYGI

*CqGPRop8* VALVTISLWFMAWTPYLIINYTG-LATIWGSLFAKANAVYNPIVYGI

*CqGPRop13* VALVTISLWFMAWTPYLIINYTG-LATIWGSLFAKANAVYNPIVYGI

*CqGPRop10* ---------------YLIINYTG-LATIWGSLFAKANAVYNPIVYGI

Figure S2.


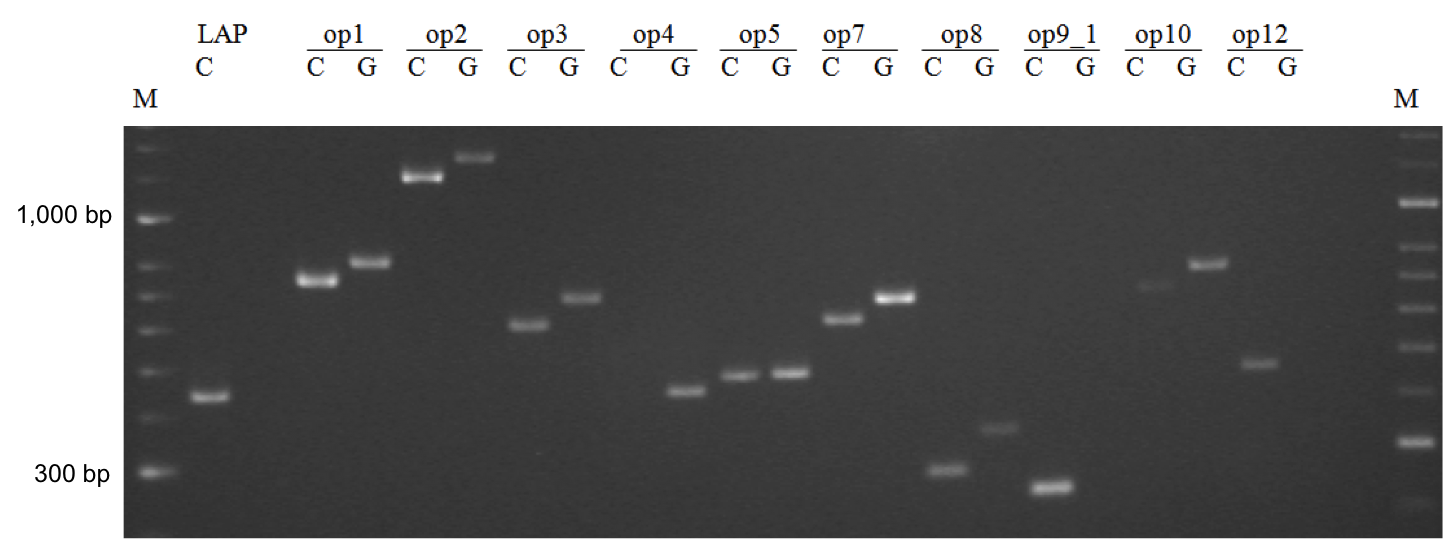


**Figure S3.**

Figure S4.

**References**

1. Brown GR, Hem V, Katz KS, Ovetsky M, Wallin C, Ermolaeva O, Tolstoy I, Tatusova T, Pruitt KD, Maglott DR *et al*. Gene: a gene-centered information resource at NCBI. Nucleic Acids Res. 2015;43:D36-42.

2. Wingender E. The TRANSFAC project as an example of framework technology that supports the analysis of genomic regulation. Brief Bioinform. 2008;9:326-332.

3. Velarde RA, Sauer CD, Walden KK, Fahrbach SE, Robertson HM. Pteropsin: a vertebrate-like non-visual opsin expressed in the honey bee brain. Insect Biochem Mol Biol. 2005;35:1367-1377.

4. Colbourne JK, Pfrender ME, Gilbert D, Thomas WK, Tucker A, Oakley TH, Tokishita S, Aerts A, Arnold GJ, Basu MK *et al*. The ecoresponsive genome of *Daphnia pulex*. Science. 2011;331:555-561.

5. Gulia-Nuss M, Nuss AB, Meyer JM, Sonenshine DE, Roe RM, Waterhouse RM, Sattelle DB, de la Fuente J, Ribeiro JM, Megy K *et al*. Genomic insights into the *Ixodes scapularis* tick vector of Lyme disease. Nat Commun. 2016;7:10507.

6. Murakami M, Kouyama T. Crystal structure of squid rhodopsin. Nature. 2008;453:363-367.

**Mosquito Opsin Protein Sequences**

***Aedes aegypti***

>AaGPRop1|AAEL006498|long wavelength sensitive opsin

MAAFVAPHFDAWQSSGNMTVVDKVPPEMLHMVHPHWNQFPPMNPLWHSILGFAIFVLGVV

SMLGNGCVIYIFTGTRSLRTPSNLLVVNLAFSDFFMMFTMGPPMVINCWHETWVFGPFAC

ELYAMFGSLFGCASIWTMTMIAFDRYNVIVKGLSAKPLTNNGALLRILGIWAFALFWTLA

PFFGWNRYVPEGNMTACGTDYLTTTWLNRSYIIVYAIFVYWTPLLTIIYSYTFILKAVSA

HEKNMREQAKKMNVASLRSSEAQQTSAEIKLAKVALVTISLWFMAWTPYLVINFTGIFKA

APISPLATIWGSLFAKANAVYNPIVYGISHPKYRAALTQKFPALSCTDAPAASNSDDNQS

TVSGATTATDEKA

>AaGPRop2|AAEL006259|long wavelength sensitive opsin

MAAFVEPHFDAWQAAGGNLTVVDKVPPEMLHMIHPHWNQFPPMNPLWHSILGFAIFVLGM

VSMIGNGFVMSIFTGTPSLRTPSNLLVVNLAFSDFLMMFTMGPPMVINCWHETWVFGPFA

CEVYACLGSLFGCASIWTMTMIAFDRYNVIVKGLAAKPLTNNGAMLRILGIWAFALFWTL

APFFGWNRYVPEGNMTACGTDYLTQTWLSRSYIIIYAIFVYWTPLLTIIYSYTFILKAVS

AHEAQMREQAKKMNVASLRSTEANQTSAEIKLAKVALVTISLWFMAWTPYLVINFTGIFK

AAPISPLATIWGSLFAKANAVYNPIVYGISHPKYRAALYQRHPWLSCQDAQESSHDNQST

VSGATTATEEKA

>AaGPRop3|AAEL006484|long wavelength sensitive opsin

MVALAEPHFQAWIQSAATNVSVVDKVPADMLHMVDAHWYQFPPMNPLWHSLLGFAIFVLC

FISLIGNGMVIYIFTNTKTLRTPSNLLVVNLAFSDFLMMFTMGPPMVYNCYHETWVLGPF

ACELYGMFGSLFGCVSIWTMTMIAFDRYNVIVKGLSAKPMGNNGALLRIFFVWGSSLAWT

LAPFFGWNRYVPEGNMSACGTDYLTDTLLSRSYILVYSIFVYFAPLLLIIYSYIFIIKAV

SAHEKNMREQAKKMNVASLRSSEAQSTSTEMKLAKVALVTISLWFMAWTPYLIINYTGIF

KAAPITPLATIWGSLFAKANAVYNPIVYGISHPKYRAALYQKFPSLSCTDAADDSQSMAS

GTTTVVQEEKPSA

>AaGPRop4|AAEL005621|long wavelength sensitive opsin

MASYGAWMAAQSAGHAVASNLTVVDRVPADMLHMVDAHWYQFPPMNPLWHSLLGFAIAVL

CFISVVGNGMVMYIFTNTKTLRTPSNLLVVNLAFSDFLMMFTMGPPMVINCYYETWVFGP

FACEVYGMFGSLFGCVSIWTMTMIAFDRYNVIVNGLSGKPLTNNGALARICGVWVSTLAW

TLAPFFGWNRYVPEGNMSACGTDYLTDTFSSRSYILVYSIFVYFAPLFLIIYSYTFIIKA

VSAHEKNMREQAKKMNVASLRSSEAQNTSTEMKLAKVALVTISLWFLAWTPYLIINYTGI

FKASPISPLATIWGSLFAKANAVYNPIVYGISHPKYRAALYQKFPSLSCTDPADDTQSVA

SGTTTVVSEKSEKTESA

>AaGPRop5|AAEL005625|long wavelength sensitive opsin

MASYGAWMAAQSAGHAVASNLTVVDRVPADMLHMVDAHWYQFPPMNPLWHSLLGFAIAVL

CFISVVGNGMVMYIFTNTKTLRTPSNLLVVNLAFSDFLMMFTMGPPMVINCYYETWVFGP

FACEVYGMFGSLFGCVSIWTMTMIAFDRYNVIVNGLSGKPLTNNGALARICGVWVSTLAW

TLAPFFGWNRYVPEGNMSACGTDYLTDTFSSRSYILVYSIFVYFAPLFLIIYSYTFIIKA

VSAHEKNMREQAKKMNVASLRSSEAQNTSTEMKLAKVALVTISLWFLAWTPYLIINYTGI

FKASPISPLATIWGSLFAKANAVYNPIVYGISHPKYRAALYQKFPSLSCTDPADDSQSVA

SGTTTVVSEKSEKTESA

>AaGPRop7|AAEL007389|long wavelength sensitive opsin

MTYYGPPPNLWGHSVTNLTVVDKVPPEIMHLVDPHWYQFPPMNPLWHSIIGFAIFMLGMI

STVGNGVVIYIFSTEKSLRTPSNLFVVNLAVSDFLMMATNATTMVYNCWFETWSLGLLMC

DLYAFTGSLFGCCSIWSMTMIAMDRYNVIVKGLSGKPLTNTGAIVRICVCWTIGIVWGSM

PMLGWNRYVPEGNMTACGTDYLTDDWFSKSYILCYSFCVYYIPLFTIIYCYVYIVKAVTV

HERTMREQAKRMNIQSLRQGDDGKAAEMKLAKIALVTISLWFLAWTPYTIINYTGVFKMA

SLTPLATIWGSVFAKCSSVYNPIVYGISHPKYRAALVRRFPALGCGDASGGADAKSMASE

VSAVSGGGSTGAGMETTAA

>AaGPRop8|AAEL009615|ultraviolet wavelength sensitive opsin

MPFEEHLSDNFTAVLRPEARLSAETRYLGWNVAPEDLPHIPEHWLKYPEPEASLHYLLGL

LYIAFTIFALVGNGLVIWVFSSAKSLRTPSNVFVVNLAICDFLMMVKTPIFIYNSFTKGF

TTGFLGCQVFAFIGSLSGIGAGATNACIAYDRYNTIARPFEGKLTHTKAIFITCMVWTYT

FPWAVLPLMELWGRFVPEGYLTSCTFDYLTNTFDNRMFVGTIFTFSYVLPMSLIIYYYSQ

IVSHVVNHEKALREQAKKMNVDSLRSNQNQANTSVEVRIAKAAITVCFLFVASWTPYAVL

ALIGAFGDKTLLTPGVTMFPACACKFVACLDPYVYAISHPRYRVELQKRLPWLAITESLP

SDSASNVTDATTANTSAPASS

>AaGPRop9_1|AAEL003035|short wavelength sensitive opsin

MFLLNETDAAIFPMARTGDMPKMLGWNLPPEQQHLVHDHWKDFPAPPYYMHLLLAMLYFV

LMSVSLIGNGIVVWIFSTSKSLRNGSNMFVVNLAIFDLLMMCEMPMFLVNSFAGYLVGYE

TSCAVYAALGSLSGIGGSITNAVIAYDRYRTISNPLDGRLNRVQSGILIFITWLWAMPFT

ILPVFKIWGRYIPEGFLTTCSFDYLTDDSDTRVFVGCIFAWAYAIPMVLICYYYGRLFGH

VSKHELMLKNQARKMNVESLASNRNEKAQSVEIRIARAAFTIFFLFVCAWTPYAIVAMIG

AYGDRTLLTPFFTMIPAVCCKIVSCLDPWVYAISHPKYRQELERRLPWMGIREPADNVST

TDSKHTVVSESLPVGPNGID

>AaGPRop_2|AAEL003035|short wavelength sensitive opsin

MFLLNETDAAIFPMARTGDMPKMLGWNLPPEQQHLVHDHWKDFPAPPYYMHLLLAMLYFV

LMSVSLIGNGIVVWIFST

>AaGPRop10|AAEL005322|unknown wavelength sensitive opsin

MRKWNAQFKSLRTPANYLVINLAIADFIIMLEAPLFVYNSYHQGPATGNVWCTIYALLGA

VGGTVAIVTLTMISIDRYNVVVYPLNPKRSTTRLKVALMIVFAWIYGLVFSVIPALDIGL

SRYTPEGFLTACSFDYLERTRDARLFMFLYFIFAWVVPIIAITFCYIQILRVVIGANSIQ

SSKNKSKTEVKLAGVVIGIIGLWFIAWTPYAIVAMMGVFGYESLLSPLGSMVPAILAKTA

ACIDPYFYAMNHPRYRQELRKMFGLNQQDLGNSQYQTSRYTRNASRMDDSEGGASERVTI

GRQPGKTTTDEPEPSQQTEQGPQPTYSKNLAANSRGALQRAQSSISAADDTSLSVSIDLT

ETNPNSNH

>AaGPRop12|AAEL005373|pteropsin

MESWAYVASAVTLFFIGFFGFFLNLFVIALMCKDVQLWTPINIILFNLVCSDFSVSIIGN

PFTLTSAISRHWIFGRTVCIAYGFFMSLLGITSITTLTVLSYERFCLISHPFSSRSLSRR

GAVFAILFIWSYSFALTSPPLFGWGAYVNEAANISCSVNWESQTLNATSYIIFLFVFGLV

VPLVVIVYSYTNIVVNMKRNAARVGRINRAEKRVTRMVFVMVLAFMIAWTPYAVFALIEQ

FGPTDIISPALGVLPALIAKSSICYNPIIYVGMNTQFRAAFNRVRNNESVDNNTITNQKD

ITMNTSKEIVECSFDFCRKKRLKIKLQSNAKSKNNNNNSRNQSIADPSSTSNGDDLDQPS

PAQTVLNSTVANSGSVASFGPKKRLRSDFELSVISSGKSILIKSNTFRSNLV

***Anopheles gambiae***

>AgGPRop1|AGAP013149|long wavelength sensitive opsin

MAAFVEPHFDAWTQGSGNMSVVDKVPPEMLHMVHPHWNQFPPMNPLWHSILGFAIFMLGM

ISMTGNGCVMYIFTNTKSLRTPSNLLVVNLAFSDFFMMFTMGPPMVINCWHETWTFGPFA

CELYAMLGSLFGCASIWTMTMIAFDRYNVIVKGLAGKPMTNNGALLRILGVWVFALFWTL

APLFGWNRYVPEGNMTACGTDYLTQTWLSRSYIIIYAIFVYWLPLLTIIYSYTFILKAVS

AHEKNMREQAKKMNVASLRTQEAQNTSTEMKLAKVALVTISLWFMAWTPYLVINFTGIFK

AAPISPLATIWGSLFAKANAVYNPIVYGISHPKYRAALYQKFPSLSCQDAPVDDGQSVAS

GATQASDEKA

>AgGPRop3|AGAP012982|long wavelength sensitive opsin

MAAFVEPHFDAWTQSGGNMSVVDKVPPEMLHMVHPHWNQFPPMNPLWHSILGFAIFMLGM

ISMTGNGCVMYIFTNTKSLRTPSNLLVVNLAFSDFFMMFTMGPPMVINCWHETWTFGPFA

CELYAMLGSLFGCASIWTMTMIAFDRYNVIVKGLAGKPMTNNGALLRILGVWVFALFWTL

APLFGWNRYVPEGNMTACGTDYLTQTWLSRSYIIIYAIFVYWLPLLTIIYSYTFILKAVS

AHEKNMREQAKKMNVASLRTQEAQNTSTEMKLAKVALVTISLWFMAWTPYLVINFTGIFK

AAPISPLATIWGSLFAKANAVYNPIVYGISHPKYRAALYQKFPSLSCQDAPVDDGQSVAS

GATQASDEKA

>AgGPRop4|AGAP012985|long wavelength sensitive opsin

MAAFVEPHFDAWTQSGGNMSVVDKVPPEMLHMVHPHWNQFPPMNPLWHSILGFAIFMLGM

ISMTGNGCVMYIFTNTKSLRTPSNLLVVNLAFSDFFMMFTMGPPMVINCWHETWTFGPFA

CELYAMLGSLFGCASIWTMTMIAFDRYNVIVKGLAGKPMTNNGALLRILGVWVFALFWTL

APLFGWNRYVPEGNMTACGTDYLTQTWLSRSYIIIYAIFVYWLPLLTIIYSYTFILKAVS

AHEKNMREQAKKMNVASLRTQEAQNTSTEMKLAKVALVTISLWFMAWTPYLVINFTGIFK

AAPISPLATIWGSLFAKANAVYNPIVYGISHPKYRAALYQKFPSLSCQDAPVDDGQSVAS

GATQASDEKA

>AgGPRop5|AGAP001162|long wavelength sensitive opsin

MMDHRPVGIFGPKSPQALTWTISVANLTVVDKVPPEMLHLVDTYWYQFPPLETKWHAALA

TTIGLLALISIVGNGCVILIFSSTKGLRTPSNLMVINLAFADFMMMFTMAPPLIINSYHE

TWVFGPLMCEIYGMFGSLSGCVSIWSMTMIAFDRYTVIVKGLSAKPLTYVGSVMRILFVW

ANSLVWTLAPLFGWNRYVPEGNMSACGTDYLSKDWISVSYIYAYSVFVYWLPLLLIIYCY

TYILKAVSAHERNMREQAKKMNVASLRSSDASKTNAEIKLAKVALVTITLWFMAWTPYLV

INYAGIIDSSPISPLQTIWGSVFAKANAVYNPIVYGISHPKYRAALYKRFPALQLRKENA

TDQNSVASCTTAADQCET

>AgGPRop6|AGAP001161|long wavelength sensitive opsin

MAAFAEPHFSAWTQTVVSNVTVVDKVPPEMLHMVDAHWYQFPPMNPLWHSILGFAIFVLG

VVSIIGNGCVIYIFTNTKALRTPSNLLVVNLAFSDFLMMFTMAPPMVINCWHETWVFGPF

ACELYAMLGSLFGCASIWTMTMIAFDRYNVIVKGLAGKPMTNNGALLRILGIWAFSLLWT

LAPLFGWNRYVPEGNMTACGTDYLSQDFTSRSYILIYSGFVYYLPLFSIIYSYIYIIQAV

SAHEKNMREQAKKMNVASLRSQEAQNTSTEMKLAKVALVTISLWFMAWTPYLVINYTGIF

KAAPISPLATIWGSLFAKANAVYNPIVYGISHPKYRAALYQKFPSLSCQDNSDDGQSVAS

AATGVSEEKPAA

>AgGPRop7|AGAP002462|long wavelength sensitive opsin [

MPYYGPMQQPGLWGQPVANLTVVDKVPPEIMHLVDPHWSQFPPMNPLWHSIIGFVIFVLG

VVSIIGNGMVIYIFSTAKSLRTPSNLFIVNLALSDFLMMGTNAFTMVYNCWFETWSLGLL

MCDLYAFFGSLFGCCSIWTMTMIALDRHNVIVHGLSGKPLTNTGAILRILLCWLIGVVWG

ILPMLGWNRYVPEGNMTACGTDYLTDDWFHKSYILVYSVFVYYTPLFTIIYAYFFIIKAV

SAHEKNMREQAKRMNVQSLRSSDDGKSTEMKLAKVALVTISLWFMAWTPYTVINYTGVFK

TASITPLATIWGSVFAKANAVYNPIVYGISHPKYRAALLRRFPSLACSDGPPADDKSLAS

EASGITSAGNPTTA

>AgGPRop8|AGAP006126|ultraviolet wavelength sensitive opsin

MGLVQLDNQTAYRPEALIGADQSGLRYLGWNVPPEELVHIPEHWLQFPEPEASLHYLLGL

LYIAFTIFSLVGNGLVIWIFIAAKSLRTPSNVFVINLAICDFFMMAKTPIFIYNSFTKGF

TLGNLGCQIFGFVGSLTGIGAGATNALIAYDRYNTITRPFEGRLTQTKAIIFICLIWAYT

IPWGVLPLLEIWGRYVPEGFLTSCTFDYLSGTFDTRLFVASIFTFSYVLPMSLIIYYYSQ

IVSHVVNHEKSLREQAKKMNVESLRSNQNQKDASVEIRIAKAAITVCFLFVASWTPYAVL

ALIGAFGDKSLLTPGVTMFPACACKFVACLDPYVYAISHPRYRIELQKRLPWLAITETLP

AENASTCTEQQDGNATTQS

>AgGPRop9|AGAP010089|short wavelength sensitive opsin

MFLGNESISEGAMLMPMARTAGEMPKLLGWNLPPEEQYLVHDHWKGFPSPPYYMHLMLAM

IYFVLMNTSLIGNGIVLWIFGTSKSLRNGSNMFIINLAIFDLLMMCEMPMFLVNSFSERL

VGYGVGCSVYAALGSMSGIGGAISNAVIAFDRYRTISNPLDGRLSRVQAGLLICLTWLWT

MPFTLLPLFEIWGRYIPEGYLTTCSFDYLTDDPDTRVFVGCIFTWAYVIPMIFICYFYAR

LFGHVRQHEMMLKNQARKMNVESLTANRSEKAQAVEMRIAKAAFTIFFLFVCAWTPYAIV

TMIGAFGDRTMLTPFVTMVPAVCCKIVSCLDPWVYAISHPKYRQELERRLPWMGIKEADD

SVSTTES

>AgGPRop10|AGAP007548|unknown wavelength sensitive opsin

MGRQGSGNAVRISPSSRNQPYFSSAHLSFVVPFPVHSKYVVRSGYVLPVDPLFVAKINPF

WLRFDPPSAGEHYGLAVFYFLMMLFGVIGNALVVFMFYRYRSLRTPANYLVINLAVADFI

IMMEAPMFIYNSIHQGPALGSIGCTVYALMGAVGGTVAIATLTVISIDRYNVVVYPLNPN

RSTTKLKCYFLIAFTWAYGLLFASFPALEIGLSRYTAEGYLTACSFDYLDRTYKARVFMF

VYFVFAWLIPFAIISYCYARILIAVINANAIQSSKSKNKTEVKLAGVVVGIIGLWFAAWT

PYAVVAMMGVFGYEQYLTPLNSMIPAVFAKIAASIDPYFYAMNHPRYRQMLERMFCNRGA

DQGNSQYQTSHYTRGASRGGDSEGGGGEESGGGGGVGRAPGGGNAGLGRGGTVRGGGGGG

RLIAGKGGGGANATGSTGGGGVKALKKQISNGDETSLEVSLEM

>AgGPRop11|AGAP002443|pteropsin

MPGRFTPCTDHRWQDVAPKMYDVTDAAAINSDHQELMAPWAYNGAAVTLFFIGFFGFFLN

IFVIALMYKDVQLWTPMNIILFNLVCSDFSVSIIGNPLTLTSAISHRWLYGKSICVAYGF

FMSLLGIASITTLTVLSYERFCLISRPFAAQNRSKQGACLAVLFIWSYSFALTSPPLFGW

GAYVNEAANISCSVNWESQTANATSYIIFLFIFGLILPLAVIIYSYINIVLEMRKNSARV

GRVNRAERRVTSMVAVMIVAFMVAWTPYAIFALIEQFGPPELIGPGLAVLPALVAKSSIC

YNPIIYVGMNTQFRAAFWRIRRSNGVAGQPDSNNTNNSNRDKESARHTAKEGLECSLDFC

HWTVRGTRVSISSAERNVPAPAARERSGGHSVTGSREESRDRHVTLKTMLSVGPRSPSSV

APVAADCSTTDVPTSGDGSVRIVRQDSELSVIHDGGGGGGGSSSRVLVIKSQKPRSNML

>AgGPRop12|AGAP002444|pteropsin

MNDAPNDVAASAVDYEDLMAPWAYNASAVTLFFIGFFGFFLNLFVIALMCKDMQLWTPMN

IILFNLVCSDFSVSIIGNPLTLTSAISHRWIFGRTLCVAYGFFMSLLGITSITTLTVLSY

ERYCLISRPFSSRNLTRRGAFLAIFFIWGYSFALTSPPLFGWGAYVQEAANISCSVNWES

QTKNATTYIIFLFVFGLVVPLIVIVYSYTNIIVNMRENSARVGRINRAEQRVTSMVAVMI

VAFMVAWTPYAIFALIEQFGPPELIGPGLAVLPALVAKSSICYNPIIYVGMNTQFRAAFS

RVRNKGQQAAADQNTTTMQRELTKSSRDMVECSFDFCRKKSRFKISLVKPTAPLAVVDVS

STSHRDKGTSRSPLDQTVLNETNEDVGRERSGGGGGGGAYAGTRFVRPDFELSVINSGKS

ILIKSKNFRSNLL

***Culex quinquefasciatus***

>CqGPRop1|CPIJ004067|long wavelength sensitive opsin

MSYYGPPPSIWGHPISNMTVVDKVPPEILHLVDPHWYQFPPMNPLWHSIIGFAIFVLGII

SVIGNGMVIYIFSTAKGLRTPSNLFVVNLAMSDFLMMMTNAFTMVYNCWYETWQLGVLMC

DLYAFTGSLFGCCSIWTMTMIALDRYNVIVKGLAGKPLTNSGAVLRILVCWTIGVVWGIL

PMLGWNRYVPEGNMTACGTDYLTEDWFHKSYILAYSFFVYYVPLFTIIYSYVYIVKAVSE

HEKSMRDQAKRMNMQSLRQGDDGKAAEMKLAKIALVTISLWFMAWTPYTIINYTGVFKTA

NISPLATIWGSVFAKANAVYNPIVYGISHPKYRAALYKRFPSLSCQDVVAADDKSLASEA

SAVTTSNSGDTASA

>CqGPRop2|CPIJ005000|short wavelength sensitive opsin

MFLLNETDAVLLPAARTGGEMVKLLGWNLPPEQMHLVHEHWKDFPAPPYFMHLLLALIYF

VLMNVSLIGNGIVVWIFTTSKSLRNGSNMFIVNLAIFDLLMMCEMPMFLVNAFSERLVGY

ETGCAIYAALGSVSGIGGAISNAVIAYDRYRTISNPLEGRMNRTKASLFVVMTWLWTVPF

TVMPMFNIWGRYIPEGYLTTCSFDYLTDDSDTRVFVGCIFAWAYAIPMVFICYFYTRLFG

HVRQHENMLKNQARKMNIESLAANRNANAEAAEIRIAKAAFTIFFLFVCAWTPYAFVAMI

GAFGDKTILTPFFTMIPAMCCKIVSCLDPWVYAISHPRYRQELEKRLPWLGIKEAPDNVS

TTESKQTVVAEPAANAET

>CqGPRop3|CPIJ009246|ultraviolet wavelength sensitive opsin

MPFLEHLSDNYTAVLRPEARLSAETRYLGWNVAPEDLPHIPEHWLKYPEPEASLHYLLGL

LYIAFTIFALVGNGLVIWIFSSAKSLRTPSNVFIVNLAICDFLMMVKTPIFIYNSFTRGF

TTGYLGCQIFAFIGSLSGIGAGATNACIAYDRYNTIARPFEGKLTHTKAIFITCLVWAYT

IPWGLLPLMEIWGRFVPEGYLTSCTFDYLTNTFDNRMFVGTIFTFSYVLPMSLIIYYYSQ

IVSHVVNHEKALREQAKKMNVDSLRSNQNQANSSVEVRIAKAAITVCFLFVASWTPYAVL

ALIGAFGDKSLLTPGVTMFPACACKFVACLDPYVYAISHPRYRVELQKRLPWLAITETLP

SDTASTTTEATTTNATASS

>CqGPRop4|CPIJ011419|unknown wavelength sensitive opsin

MPQDCTQTTKCGILHPASQEIPWKWKGKDQVLEVLLQIFSWTTSVVCGCRMWMASTASTA

DLTGGGYVAYGRDLHRSWELKGKDHQLCWSEPGFEPRSTAYETEALPLGYVARFKSLRTP

ANYLVINLAVADFLIMLEAPIFVYNSYHLGPAFGNTLCTIYSLLGAIGGTVAIMTLTMIS

VDRYNVVVYPLNPNRSTTRLKVMLMIVFTWIYALVFSLMPALEIGLSRYTPEGFLTACSF

DYLDRGWDARVFMFMYFVFAWVIPFLTISYCYVAILRVVVGAGSIQSSKNKNKQEVKLAG

VVIGIIGLWFIAWTPYAVVAMLGVFGYEHLLTPLGSMIPAILAKTASCIDPYFYAMNHPR

FRQELRKMFGKEQEMNHSQYQTSRYTRNASRNDSEAGPSERVQLGRAPGKDADPIPAVSS

SVAQPNYSQNLASNRKGGLQRAQSSISAADDTSLSCSIDLTETQPNNH

>CqGPRop5|CPIJ012052|long wavelength sensitive opsin

MAAFAEPHFDAWQAGAGNMSVVDMVAPEMLHMVHPHWNQFPPMNPLWHSILGFAIFCLGM

VSMIGNGCVISIFTGTKSLKTPSNLLVVNLAFSDFLMMFTMGPPMVINCWHQTWVFGPFA

CELYACLGSLFGCASIWTMTLIAFDRYNVIVKGLAAKPMTNSGAMVKILMVWAFALFWTL

APFFGWNRYVPEGNMTACGTDYLTQTWLSRSYIIVYAIFVYWLPLLTIIYSYTFILKAVS

AHEEQMREQAKKMNVASLRSSEAQQTSAEIKLAKVALMTISLWFMAWTPYLVINFTGIFK

AAPISPLATIWGSLFAKANAVYNPIVYGISHPKYRAALYQRYPGLSCQSDNSSNDSQSVA

TTAEEEKA

>CqGPRop6|CPIJ011571|long wavelength sensitive opsin

MAAFVEPHFDAYQAGNGNMTVVDMVSPDMLHMVHPHWNQFPPMNPLWHSILGFAIFCLGM

VSMIGNGCVISIFTSTKSLKTPSNLLVVNLAFSDFLMMFTMGPPMVINCWHQTWSFGPFA

CELYACLGSLFGCASIWTMTLIAFDRYNVIVKGLAAKPMTNSGAMVKILLVWAFALFWTL

APFFGWNRYVPEGNMTACGTDYLTQTWLSRSYIIVYAVFVYWLPLLTIIYSYTFILKAVS

AHEEQMREQAKKMNVASLRSSEAQQTSAEIKLAKVALVTISLWFMAWTPYLVINFTGVFK

AAPITPLATIWGSLFAKANAVYNPIVYGISHPKYRAALYQRYPGLSCQSDNSSNDTQSVA

SGATTASDEKA

>CqGPRop7|CPIJ011573|long wavelength sensitive opsin

MLGEPSLPMALSWGSGPVSNMTVVDKVLPDMLHLVDAHWYQFPPMNPLWHSILGFAIFVL

GCVSVIGNGCVVSIFTGTKSLRTPSNMLVINLAFSDFLMMFTMAPPMVMSCYHETWVFGP

LMCQVYAMLGSLFGCVSIWSMTMIAFDRYNVIVKGLSATPMTFNNALLKIAFIWANALIW

TLAPMFGWNRYVPEGNMTACGTDYLTQDIVSTSYIMVYSLFVYWLPLLMIIYSYTFILKA

VADHEKNMREQAKKMNVASLRSSDAAKQSAEIRLAKVALVTISLWFLAWTPYLVINYTGI

FKAAPISPLATIWGSLFAKANAVYNPIVYGISHPKYRAALYKKYPSLACSTDSVSDDQSV

ASGATVASDNNNA

>CqGPRop8|CPIJ011574|long wavelength sensitive opsin

MASYAAWNAAQSGLGAMVNLTVVDRVPADMLHMVDAHWYQFPPMNPLWHSILGFAIFVLC

FISLVGNGCVVYIFTNTKTLRTPSNLLVVNLAFSDFLMMFTMGPPMVINCYHETWVFGPF

ACELYGMLGSLFGCVSIWSMTLIAFDRYNVIVKGLSAKPMTNNGALLRIFLVWASCLAWT

IPPFFGWNRYVPEGNMSACGTDYLTDTFLSRSYILVYSVFVYFAPLLMIIYSYIFIIKAV

SAHEKNMREQAKKMNVASLRSSEAQNTSTEMKLAKVALVTISLWFMAWTPYLIINYTGIF

KAAPITPLATIWGSLFAKANAVYNPIVYGISHPKYRAALYQKFPSLSCTDAVDDSQSVTS

GTTTVVNEEKPSA

>CqGPRop9|CPIJ011576|long wavelength sensitive opsin

MASYAAWNAAQSGLGAMVNLTVVDRVPADMLHMVDAHWYQFPPMNPLWHSILGFAIFVLC

FISLVGNGCVVYIFTNTKTLRTPSNLLVVNLAFSDFLMMFTMGPPMVINCYHETWVFGPF

ACELYGMLGSLFGCVSIWSMTLIAFDRYNVIVKGLSAKPMTNNGALLRIFLVWASCLAWT

IPPFFGWNRYVPEGNMSACGTDYLTDTFLSRSYILVYSVFVYFAPLLMIIYSYIFIIKAV

SAHEKNMREQAKKMNVASLRSSEAQNTSTEMKLAKVALVTISLWFMAWTPYLIINYTGIF

KAAPITPLATIWGSLFAKANAVYNPIVYGISHPKYRAALYQKFPSLSCTDAVDDSQSVTS

GTTTVVNEEKPSA

>CqGPRop10|CPIJ013056|long wavelength sensitive opsin

MLISEVTCNMASYAAAWKAAEAVANLTVVDKVPADMLHMVDAHWYQFPPMNPLWHAMLGW

AIFFLCLISVIGNGMVVYIFTNTRTLRTPSNLLVVNLAFSDFLMMFTMGPPMVINCYYET

WVFGAFACEVYGMFGSLFGCVSIWTMTMIAFDRYNVIVKGLSAKPLTNNGAMVQIFGVWI

ASLGWTLAPFLGWNRYVPEGNMSACGTDYLTDTMLSRSYILVYSMFVYFAPLLLIIYSYI

FIIKAVSAHEKNMREQAKKMNVASLRSQETQSTSTEMKLPSRAVTISSGLWLGRAAPITP

LATIWGSLFAKANAVYNPIVYGISHPKYRAALYQKFPSLSCSDSVEDTQSMASAETTVAE

KTAA

>CqGPRop11|CPIJ013408|short wavelength sensitive opsin

MFLVNETDAVLLPAARTGEMVKLLGWNLPPEQMHLVHEHWKDFPAPPYFMHLLLALIYFV

LMNLSLIGNGIVVWIFTTSKSLRNGSNMFIVNLAIFDLLMMCEMPMFLVNAFSERLVGYE

TGCAIYAALGSVSGIGGAISNAVIAYDRYRTISNPLEGRMNRVQASLFVLLTWLWTLPFT

VLPMFNIWGRYIPEGYLTTCSFDYLTDDSDTRVFVGCIFTWAYAIPMVFICYFYTRLFGH

VRQHENMLKNQARKMNIESLAANRNVNAEAAEIRIAKAAFTIFFLFVCAWTPYAIVTMIG

AFGDKTMLTPFFTMVPAMCSKVVSCLDPWVYAISHPKYRQELERRLPWLGIKEAPDNVST

TESKQTVISDPPAAAEN

>CqGPRop12|CPIJ014334|pteropsin

MPVAGVDHWNWPPPGLEWPEAPRMFRWGDICRTLWTPMNIILLNLVCSDFSVSIVGNPFT

LSSAISHRWLFGRKLCVAYGFFMSLLGITSITTLTVLSYERFYLISRPFSSRSLSRRGAL

GAVLLIWCYSFALTSPPLFGWGAYVNEAANISCSVNWETQTLNATTYIIYLFVFGLVVPL

TVIVYSYTNIIVNMKKNAARVGRINRAEKRVTTMVAVMVIAFMVAWTPYSVFALMEQFGP

PDVIGPGLAVLPALIAKSSICYNPIIYVGMNTQFRAAFNRVRNHDPGDMANTTTNQKELT

RSSRDVASVDCSFDFCRKKNRLKMKLHNSIHRSAAIKAGRSQSPHSESERSSSGATQERS

TRVQTMLNATVDSRSISGSTGKLMKSDFELSVINSGKSILIKSNTFRSNLV

>CqGPRop13|CPIJ020021|long wavelength sensitive opsin

MASYAAWTAVKAGVGAAMNLTVVDKVPADMLHMVDAHWYQFPPMNPLWHAMLGWAIFFLC

LISLIGNGMVINIFTSTKTLKTPSNLLVVNLAFSDFLMMFTMGPPMVMNCYYETWVFGPF

ACEVYAMCGSLFGCISIWTMTMIAFDRYNVIVNGISAEPLTNKGATIRIFAIWATSFAWT

LAPFFGWNRYVPEGNMSACGTDYLTDTFSSRSYILMYSVFVYFAPLFLIIYSYIFIIKAV

AAHEKNMREQAKKMNVASLRSSEAQNTSTEMKLAKVALVTISLWFMAWTPYLIINYTGIF

KAAPITPLATIWGSLFAKANAVYNPIVYGISHPKYRAALYKKFPALSCTDSADDSQSVAS

GETVITEKAEKIEA
